# Heterogeneous Reactions of N_2_O_5_ with Nitrate- and Chloride-Containing Solutions: Isotopic Evidence for the Nitration of N_2_O_5_

**DOI:** 10.1021/acs.jpca.5c06048

**Published:** 2025-11-10

**Authors:** Thomas F. Derrah, Pascale S. J. Lakey, Steven J. Kregel, Manabu Shiraiwa, Gilbert M. Nathanson, Timothy H. Bertram

**Affiliations:** † Department of Chemistry, 5228 University of Wisconsin − Madison, Madison, Wisconsin 53706, United States; ‡ Department of Chemistry, 8788 University of California − Irvine, Irvine, California 92697, United States

## Abstract

The nitrate (NO_3_^–^) ion has been shown to suppress the reactive uptake of dinitrogen pentoxide (N_2_O_5_) to aqueous aerosol, yet a molecular mechanism that explains this effect remains elusive. To explore how N_2_O_5_ and NO_3_^–^ interact in solution, we use isotopically labeled ^15^NO_3_^–^ in the presence of Cl^–^ to mark ^14,14^N_2_O_5_ that react with ^15^NO_3_^–^ by detecting the nitration and chlorination products ^14,15^N_2_O_5_, ^15,15^N_2_O_5_, Cl^14^NO_2,_ and Cl^15^NO_2_. At three Na^15^NO_3_ concentrations (0.47, 1.35, and 3.7 M), both Cl^14^NO_2_ and Cl^15^NO_2_ products are measured as the NaCl concentration increases from 0 to 3.0 M, confirming that N_2_O_5_ can reactively exchange with NO_3_^–^ prior to chlorination. Using a kinetic multilayer model to compare chlorination and nitration (nitrate exchange) of N_2_O_5_, the rate constant ratio *k*_Cl^–^_ */k*_NO_3_^–^_ is determined to be between 2 and 6 for the three ^15^NO_3_^–^ concentrations. Model results suggest that the initial adsorption of a ^14,14^N_2_O_5_ molecule produces the majority of Cl^15^NO_2_ by exchange with ^15^NO_3_^–^ before reacting with Cl^–^, rather than occurring through multiple absorption and evaporation events involving ^14,15^N_2_O_5_ or ^15,15^N_2_O_5_. The ratio of nitrate exchange to hydrolysis, *k*_NO_3_^–^_ */k*_H_2_O_, is inferred to be between 70 and 230, which overestimates nitrate suppression in 6 M NaNO_3_ by 10-fold when using previous parametrizations of N_2_O_5_ uptake based on an S_N_1 NO_2_^+^ mechanism. We propose an alternate S_N_2 mechanism involving N_2_O_5_ activation and deactivation that fits both isotope and uptake data.

## Graphical abstract

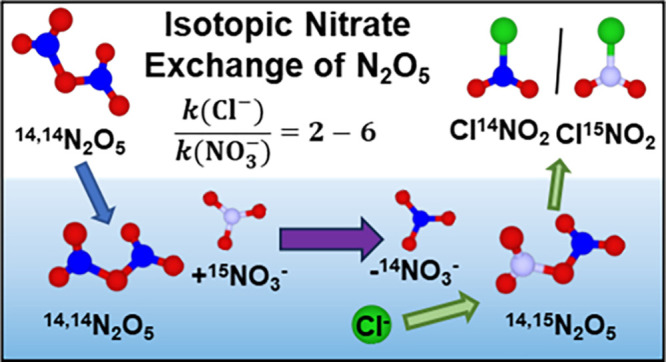

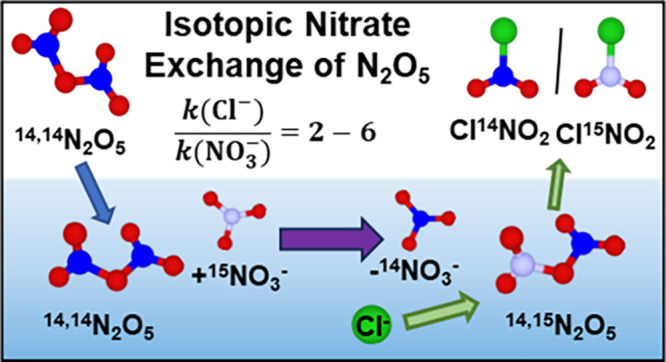

## Introduction

Reactive uptake to aqueous aerosol is the primary loss process for dinitrogen pentoxide (N_2_O_5_) and a nocturnal sink for NO_*x*_ (NO_*x*_ ≡ NO + NO_2_).^1–3^ On a global scale NO_*x*_ loss lowers tropospheric ozone concentrations,^1^ subsequently reducing hydroxyl radical concentrations and increasing the lifetimes of greenhouse gases such as methane.^2^ N_2_O_5_ can undergo several reactions depending on aerosol composition, such as hydrolysis to form nitric acid (HNO_3_) and chlorination to form nitryl chloride (ClNO_2_).^1,4^ The hydrolysis pathway leads to nitrification of aerosol and contributes to elevated aerosol mass in urban regions.^5–8^ The chlorination pathway has further implications for air quality because ClNO_2_ evaporates from aerosol particles and photolyzes into Cl and NO_2_ radicals, which in turn impact tropospheric ozone and hydroxyl concentrations.^9–13^

The probability that a N_2_O_5_ molecule will collide and react with an aerosol particle is called the reactive uptake coefficient, γ. Two major constituents that reduce the reactive uptake of N_2_O_5_ are organic films on aerosol particles^14–16^ and high (>1 M) concentrations of nitrate (NO_3_^–^).^17–22^ This latter suppression by NO_3_^–^ is called the “nitrate effect”, a phenomenon that is observed both in laboratory experiments^17–20^ and in field measurements.^21,22^ Bertram and Thornton found that NO_3_^–^ does not significantly lower the uptake coefficient until concentrations reach 0.5–1 M, and at saturated concentrations (near 7 M NaNO_3_ or NO_3_^–^: H_2_O mole ratio of 0.17) the uptake probability decreases to ∼0.005, about a factor of 6 lower than the uptake of N_2_O_5_ of ∼0.03 into pure water.^17^ Griffiths et al. further determined that the uptake coefficient decreases by 50% at 1 M NO_3_^–^, and the rate of reaction of N_2_O_5_ decreases by a factor of 7 as the NO_3_^–^:H_2_O mole ratio increases from near 0 to 0.20.^18^ Wahner et al. also found a lower N_2_O_5_ uptake in very concentrated NaNO_3_ aerosol particles: at 50 and 60% relative humidity and 13.5 and 10.6 M NaNO_3_, the measured uptake is 0.0018 and 0.0032, respectively.^20^

Previous investigators have hypothesized a mechanism for N_2_O_5_ reactivity involving the dissociation of a solvated N_2_O_5_ molecule into a nitrate anion, NO_3_^–^, and a nitronium cation, NO_2_^+^ ([Disp-formula eqR1] and [Disp-formula eqR2]).^20,23,24^ NO_2_^+^ can then react with H_2_O to form H^+^ and another NO_3_^–^ or react with other aqueous species such as Cl^–^ to form ClNO_2_ ([Disp-formula eqR3] and [Disp-formula eqR4]):

$$N_{2}O_{5}\;(g)↔N_{2}O_{5}\;(\mathrm{aq}),\mathrm{dissolution}\;\mathrm{and}\;\mathrm{evaporation}$$ R1

$$N_{2}O_{5}\to {{\mathrm{NO}}_{2}}^{+}+{{\mathrm{NO}}_{3}}^{-},\mathrm{ionization}\;\mathrm{and}\;\mathrm{separation}$$ R2

$${{\mathrm{NO}}_{2}}^{+}+{{\mathrm{NO}}_{3}}^{-}\to N_{2}O_{5},\mathrm{recombination}$$ R-2

$${{\mathrm{NO}}_{2}}^{+}+H_{2}O\to 2H^{+}+{{\mathrm{NO}}_{3}}^{-},\mathrm{hydrolysis}$$ R3

$${{\mathrm{NO}}_{2}}^{+}+{\mathrm{Cl}}^{-}\to {\mathrm{ClNO}}_{2},\mathrm{chlorination}$$ R4

The S_N_1-type mechanism above was first proposed by Mozurkewich and Calvert, based on prior research on N_2_O_5_ dissociating into ions in neat nitric acid.^23^ This reaction pathway is effective in explaining the measured kinetics from a range of experiments.^17,18,20,23,25^ In this mechanism, the reactive uptake of N_2_O_5_ is controlled by the rate-limiting step [Disp-formula eqR2] to form the reactive NO_2_^+^ ion. Because this step is rate limiting, the N_2_O_5_ uptake coefficient appears largely independent of the concentration of reactive partners such as Cl^–^. This independence is in accord with previous studies, which do not find a measurable change in N_2_O_5_ uptake into aerosol or NaCl solutions up to 5 M.^24,26–28^ The nitrate effect is attributed to the second-order reaction of added NO_3_^–^ with NO_2_^+^ to reform N_2_O_5_ ([Disp-formula eqR-2]), leading to a reduction in the amount of N_2_O_5_ irreversibly lost to hydrolysis or chlorination. As expected from the competition between [Disp-formula eqR-2] and [Disp-formula eqR4], the addition of Cl^–^ to NO_3_^–^ aerosol can overcome the nitrate effect, increasing the uptake coefficient back to ∼0.03 at a Cl^–^/NO_3_^–^ mole ratio > 0.2.^17^

Recent computational studies bring into question the existence of solvent-separated NO_2_^+^ ions in the mechanism described above. Through ab initio molecular dynamics (AIMD) simulations of N_2_O_5_ reacting with ions in water clusters, Karimova et al. and Molina and Gerber did not locate an independently solvated NO_2_^+^ species.^29,30^ Rather, they predict that N_2_O_5_ exists as a contact ion pair, NO_2_^δ+^NO_3_^δ−^, close to a nearby water molecule. Further AIMD studies by Hirshberg et al.^31^ indicate that NO_2_^δ+^NO_3_^δ−^ species rapidly fluctuate in charge on the picosecond time scale, with most probable charges on each N group of ±0.21. Galib and Limmer additionally find that the NO_2_^+^ species, in the form of H_2_ONO_2_^+^, exists only transiently with an average lifetime of 4 ps,^32^ and Feng et al. identify even shorter times.^33^

With a solvent-separated NO_2_^+^ or H_2_ONO_2_^+^ species predicted to be short-lived and therefore unlikely to be the primary mechanism by which N_2_O_5_ reacts with Cl^–^, alternative mechanisms should be considered. One option is a set of S_N_2 reactions ([Disp-formula eqR5]–[Disp-formula eqR7]) described by Karimova and Gerber,^29^ McCaslin et al.,^34^ Moon and Limmer,^35^ and Tang et al:^36^

$$N_{2}O_{5}+H_{2}O\to 2H^{+}+2{{\mathrm{NO}}_{3}}^{-},\mathrm{hydrolysis}$$ R5

$$N_{2}O_{5}+{\mathrm{Cl}}^{-}\to {\mathrm{ClNO}}_{2}+{{\mathrm{NO}}_{3}}^{-},\mathrm{chlorination}$$ R6

$$N_{2}O_{5}+{{\mathrm{NO}}_{3}}^{-}\to N_{2}O_{5}+{{\mathrm{NO}}_{3}}^{-},\mathrm{nitration}$$ R7

However, in this mechanism, there is no apparent rate-limiting step for N_2_O_5_ prior to reaction with Cl^–^. Higher concentrations of Cl^–^ should therefore increase reactive uptake of N_2_O_5_, in contrast to observations.^24,26–28^ It appears that some activated form of N_2_O_5_ created in a rate-limiting step is required to account for the insensitivity to changing Cl^–^ concentrations, but we do not have experimental evidence for the nature of this transient species. It may be in the form of an extreme fluctuation in charge of NO_2_^δ+^NO_3_^δ−^ in a particular Cl^–^/H_2_O first solvation shell, as anticipated from studies by Hirshberg et al.,^31^ or it may rely on the joint positioning of N_2_O_5_ and Cl^–^ in the interfacial region, as discovered theoretically by Limmer and co-workers.^32,35,37,38^ In particular, Moon and Limmer find that the formation of ClNO_2_ is accelerated by a charge-delocalized transition state [Cl^δ−^NO_2_^δ+^NO_3_^δ−^]^−^ near the surface.^35^ For the purposes of this kinetic study, we refer to this energetic and/or spatially localized N_2_O_5_ species as N_2_O_5_* ([Disp-formula eqR8]–[Disp-formula eqR10]). We use a single * for activated N_2_O_5_ in all reactions, although the required extent of activation or optimum location in the near-interfacial region may differ for each reaction:^35^

$$N_{2}O_{5}\to N_{2}{O_{5}}^{*},\mathrm{activation}\;(\mathrm{rate}\;\mathrm{limiting})$$ R8

$$N_{2}{O_{5}}^{*}\to N_{2}O_{5},\mathrm{deactivation}\;\mathrm{by}\;\mathrm{solvent}\;H_{2}O$$ R-8

$$N_{2}{O_{5}}^{*}+H_{2}O\to 2H^{+}+2{{\mathrm{NO}}_{3}}^{-},\mathrm{hydrolysis}\;k_{w}$$ R9

$$N_{2}{O_{5}}^{*}+{\mathrm{Cl}}^{-}\to {\mathrm{ClNO}}_{2}+{{\mathrm{NO}}_{3}}^{-},\mathrm{chlorination}\;k_{{\mathrm{Cl}}^{-}}$$ R10

Analogous fluctuations in N_2_O_5_* location or H_2_O solvation may deactivate N_2_O_5_* ([Disp-formula eqR-8]), a step that is the reverse of N_2_O_5_ activation ([Disp-formula eqR8]) This reaction scheme provides an explanation for the nitrate effect if an additional reaction is added:

$$N_{2}{O_{5}}^{*}+{{\mathrm{NO}}_{3}}^{-}\to N_{2}O_{5}+{{\mathrm{NO}}_{3}}^{-},\mathrm{nitrate‐induced}\;\mathrm{deactivation}\;k_{{\mathrm{NO}}_{3}^{-}}$$ R11

Nitrate deactivation may take at least two forms as NO_3_^–^ enters the solvent shell surrounding an N_2_O_5_* molecule. First, NO_3_^–^ may interact nonreactively to deenergize N_2_O_5_* or prohibit its activation by suppressing the ability of water molecules to stabilize a fluctuating NO_2_^δ+^/NO_3_^δ−^ charge distribution (as observed in simulations by Galib and Limmer),^32^ by disrupting a reactive Cl^–^ or H_2_O configuration,^30,32^ or by altering positions of N_2_O_5_ and/or Cl^–^ in the interfacial region, as postulated by Moon and Limmer.^35^ We note that added Cl^–^ and Na^+^ ions may also deactivate N_2_O_5_*, but this effect has not yet been identified and is complicated by irreversible reaction via [Disp-formula eqR10] (see Section IV of the Supporting Information (SI)). Second, the nitrate ion may react chemically with N_2_O_5_* by exchanging NO_3_^–^ ions, perhaps through an [NO_3_^δ−^NO_2_^δ+^NO_3_^δ−^]^−^ transition state that falls apart back into inactivated N_2_O_5_ and NO_3_^–^_._ We specifically focus in this study on exploring whether dissolved N_2_O_5_ undergoes chemical exchange with NO_3_^–^ prior to N_2_O_5_ evaporation or reaction with Cl^–^. The observation of extensive isotopic exchange would imply that the original N_2_O_5_ entering solution is not the same N_2_O_5_ that evaporates or reacts with Cl^–^ or H_2_O.

These nitration experiments require a way to chemically distinguish reactant and product N_2_O_5_ species in a nitration reaction. Inspired by the work of Gržinić, Bartels-Rasuch, Turler, and Ammann,^25,39^ who used ^13,14^N_2_O_5_ to interrogate reactive uptake, we elect to use isotopically labeled ^15^NO_3_^–^ to distinguish N_2_O_5_ molecules that do not exchange in solution from molecules that undergo a ^15^NO_3_ exchange reaction to form ^14,15^N_2_O_5_ or ^15,15^N_2_O_5_ ([Disp-formula eqR12] and [Disp-formula eqR13]). We therefore focus on measurements of isotope product yields rather than uptake measurements themselves.

Incorporating ^15^NO_3_^–^ exchange into [Disp-formula eqR11] results in three unique N_2_O_5_ species in solution, namely ^14,14^N_2_O_5_, ^14,15^N_2_O_5_, and ^15,15^N_2_O_5_:

N14,142O5*+NO153−→N14,152O5+NO143−⁣NO153−exchange R12

N14,152O5*+NO153−→N15,152O5+NO143−⁣NO153−exchange R13

These isotopic species can also undergo activation, [Disp-formula eqR8], and react with solvent H_2_O to form ^14^NO_3_^–^ or ^15^NO_3_^–^ or react with solute Cl^–^ to form Cl^14^NO_2_ and Cl^15^NO_2_ ([Disp-formula eqR14]–[Disp-formula eqR17]):

N214,15O5*+H2O→NO314−+NO315−+2H+⁣hydrolysis R14

N215,15O5*+H2O→2NO315−+2H+⁣hydrolysis R15

N214,15O5*+Cl−→12ClNO214+12NO315−+12ClNO215+12NO314−⁣chlorination R16

N215,15O5*+Cl−→ClNO215+NO315−⁣chlorination R17

We can identify the exchange of ^15^NO_3_^–^ with activated N_2_O_5_ species through measurements of the labeled nitration products ^14,15^N_2_O_5_ and ^15,15^N_2_O_5_ and chlorination products Cl^14^NO_2_ and Cl^15^NO_2_ after they evaporate. The reaction rate constants for nitrate exchange ([Disp-formula eqR11]–[Disp-formula eqR13]), hydrolysis ([Disp-formula eqR9], [Disp-formula eqR14], and [Disp-formula eqR15]), and chlorination ([Disp-formula eqR10], [Disp-formula eqR16], and [Disp-formula eqR17]) are labeled *k*_NO_3_^–^_, *k*_w_, and *k*_Cl^–^_, respectively. As we show below, we observe in the gas phase both the first generation isotopically labeled nitration product (^14,15^N_2_O_5_) and second generation isotopically labeled nitration and chlorination products (^15,15^N_2_O_5_ and Cl^15^NO_2_). The concentration of the isotopically labeled N_2_O_5_ products is determined to be small, as predicted by kinetic modeling of the flow reactor discussed in Section I of Supporting Information (SI). We focus most of our analysis instead on the chlorination products, as they are terminal products in our experiment and quickly evaporate. The measured ratio of Cl^14^NO_2_ and Cl^15^NO_2_ production rates yields the ratio of rate constants *k*_Cl^–^_ */k*_NO_3_^–^_ for chlorination and nitrate exchange that we seek. Section IV of the SI shows that, for the S_N_2 mechanism, this ratio is independent of the rates and nature of the deactivation steps [Disp-formula eqR-2], [Disp-formula eqR-8], and [Disp-formula eqR11] if N_2_O_5_* is the same energized or spatially located reactant for chlorination, nitrate exchange, and hydrolysis.

## Experimental Procedure

The production of ^14,15^N_2_O_5_, ^15,15^N_2_O_5_, Cl^14^NO_2_, and Cl^15^NO_2_ was measured from the reaction of N_2_O_5_ with solutions of 0.47, 1.35, and 3.7 M Na^15^NO_3_ containing concentrations of NaCl from 0 to 3.0 M. We followed the experimental approach of Staudt et al.^40^ and Kregel et al.,^41^ where trace amounts of N_2_O_5_ in air alternate between flowing over mixed Na^15^NO_3_/NaCl sample solutions and a saturated NaCl reference solution (5.8 M NaCl) in D_2_O. The experiments were performed at atmospheric pressure where gas-phase diffusion is slow. At this pressure, the disappearance of N_2_O_5_ or appearance of isotopic N_2_O_5_ or ClNO_2_ is not governed by the intrinsic reactivity of N_2_O_5_ in the solution, but by N_2_O_5_ gas-phase diffusion to the surface. This rate-limiting diffusion prohibits us from measuring the reactive uptake coefficient (fraction of impinging N_2_O_5_ that react upon collision from the gas phase). The relative fluxes of isotopic species evaporating from solution instead reflect the competition between chlorination and nitration of N_2_O_5_.

### N_2_O_5_ Production

N_2_O_5_ was produced following a procedure similar to the one used by Bertram and Thornton.^42^ Ultra high purity zero air (∼20% O_2_) (Airgas) and Ultra high purity N_2_ (Airgas) flowed over potassium hydroxide pellets to ensure dry conditions before the gas mixture was illuminated with a mercury pen lamp (Jelight 95-2100-1) to produce O_3_. The resulting O_3_/N_2_/O_2_ gas mixture was then combined with 50 ppm of NO_2_ mixed with N_2_ (Airgas). The gas mixture was allowed to react in a darkened reaction vessel for approximately 100 s to produce N_2_O_5_. This gas mixture was then used for experiments in the flow reactor. The final concentrations of N_2_O_5_, O_3_, NO_2_, and NO_3_ in the gas mixture over the solutions are 6, 26, 241, and 0.004 ppb. The O_3_ concentration was measured using a Personal Ozone Monitor (2B Technologies). We calculated the concentrations of N_2_O_5_ and NO_3_ using the measured initial concentrations of NO_2_ and O_3_ and published reaction rate constants.^42,43^ Variations in the synthesis system, such as the temperature of the laboratory and minor changes in the flow rates of gas species, result in the actual N_2_O_5_ concentration having small fluctuations between days. Since the product yields involve the relative production of isotopically labeled N_2_O_5_ or ClNO_2_ species, the absolute concentration of initial N_2_O_5_ did not affect the results.

Experiments probing N_2_O_5_ exchange with isotopically labeled ^15^NO_3_^–^ were performed in a lab-designed flow reactor system (Figure 1) described by Kregel et al.^41^ A Teflon vessel contained solutions of 0.47, 1.35, or 3.7 M Na^15^NO_3_ (^15^N 98%+, Cambridge Isotope Laboratories) with variable concentrations of 0 to 3.0 M NaCl (EMD Millipore) in D_2_O (99.9% D, Sigma-Aldrich). We used two solutions at the middle concentration range of Na^15^NO_3_ (1.33 and 1.36 M). Due to their similar concentrations, we present their data together as 1.35 M Na^15^NO_3_. NaCl concentrations were achieved through serial addition of small aliquots of concentrated NaCl solution to the solution vessel containing the Na^15^NO_3_ solution. A second Teflon vessel contained saturated NaCl in D_2_O; this highly concentrated solution acted as a reference because the branching fraction of ClNO_2_ from N_2_O_5_ is expected to be unity in the absence of ^15^NO_3_^–^.^40,41^ Both solutions were held at 20 °C by a temperature-controlled chiller. The N_2_O_5_ gas flow was alternated between the two solution vessels by a computer-controlled switching valve, with the N_2_O_5_ flow active for 2 min for each pass over a solution. After 4 passes over each boat, the signals of each species of interest were averaged. It is important to note that D_2_O from the boats evaporates during the course of an experiment. From previous tests, we estimate about 1 mL of D_2_O from the 20 mL solution is lost after 2 h. The serial additions of small aliquots of concentrated NaCl in D_2_O between experiments to increase the Cl^–^ concentration mitigated the net D_2_O loss from the boats.

**Figure 1:**
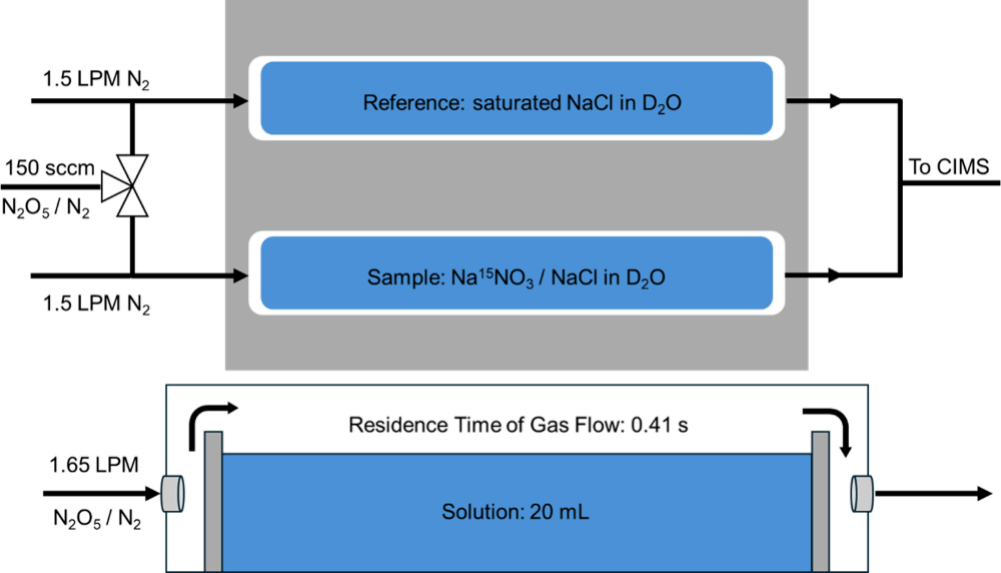
Depiction of the flow reactor setup and a side view of one of the Teflon vessels containing solution. A flow of 150 cm^3^/min (sccm) of N_2_O_5_ in N_2_ is mixed with an additional 1.5 L/min (LPM) flow of N_2_ carrier flow over the solutions. This gas flow is directed over 20 mL of a reference solution containing saturated NaCl in D_2_O or a sample solution of variable Na^15^NO_3_ and NaCl in D_2_O. The residence time of the gas over the solution is 0.41 s. The resulting gas flow enters the chemical ionization mass spectrometer (CIMS). Reproduced from ref 14. Copyright 2023 American Chemical Society.

### Reactant and Product Detection

Isotopically labeled N_2_O_5_ and ClNO_2_ evaporating from the flow reactor were measured using a quadrupole mass spectrometer. We used chemical ionization with iodide-adduct (I^–^) chemistry to detect each species. The primary peaks of interest are I­(^14,14^N_2_O_5_)^−^ (*m*/*Q* = 234.89), I­(^14,15^N_2_O_5_)^−^ (*m*/*Q* = 235.88), I­(^15,15^N_2_O_5_)^−^ (*m*/*Q* = 236.88), I­(^35^Cl^14^NO_2_)^−^ (*m*/*Q* = 207.87), and I­(^35^Cl^15^NO_2_)^−^ (*m*/*Q* = 208.86). Nitric acid was also detected in the mass spectrometer after hydrolysis of N_2_O_5_ on tubing that had become wet. Due to the mass peak overlap of I­(HNO_3_*H_2_O)^−^ (*m*/*Q* = 207.91) with I­(^35^Cl^14^NO_2_)^−^, all experiments were run in D_2_O, which raises the nitric acid peak to I­(DNO_3_*D_2_O)^−^ (*m*/*Q* = 210.93). However, this peak now overlaps with I­(^37^Cl^15^NO_2_)^−^ (*m*/*Q* = 210.86). As a result, our analysis focused only on the ^35^Cl isotopes of ClNO_2_ species, ^35^Cl^14^NO_2_ and ^35^Cl^15^NO_2_.

### Kinetic Multilayer (KM) Model

To determine the relative kinetic rates of reaction of species with N_2_O_5_, we use a kinetic multilayer model that incorporates the distinct steps involved in gas–liquid reactions: diffusion of reactant and products species through the gas phase, passage of these through the interfacial region, and diffusion and reaction of each species ([Disp-formula eqR8]–[Disp-formula eqR17]) in the liquid bulk phase.^44,45^ The gas and bulk phases are separated into multiple layers to determine the diffusion of species toward and away from the surface. Further details concerning the specific parameters for the model, including reaction rate constants, are presented in Figure S1 and Tables S1 and S2 in the Supporting Information (SI). Additionally, the SI describes an assessment of the sensitivity of the model to parameters such as the boundary layer thickness in our reactor cell in Figures S2 and S3. We find that variations in boundary layer thickness (estimated to be roughly 0.1 cm) impact the absolute amount of isotopically labeled species produced but have no effect on the ratios of products that we report here.

## Results and Analysis

We first conducted experiments to measure the production of ^14,15^N_2_O_5_ and ^15,15^N_2_O_5_ from reaction of ^14,14^N_2_O_5_ with a near-saturated 6.9 M Na^15^NO_3_/D_2_O solution. Figure 2A shows the I^–^ CIMS mass spectrum, where the height of the I­(^14,15^N_2_O_5_)^−^ peak doubles when sampling from the reactor containing the Na^15^NO_3_ solution compared to the 4.6 M NaCl solution with no ^15^NO_3_^–^. This difference highlights that a nitration reaction indeed occurs. The absolute magnitude of the ^14,15^N_2_O_5_ and ^15,15^N_2_O_5_ peak heights are small relative to the height of the ^14,14^N_2_O_5_ peak, with the ^15,15^N_2_O_5_ signal being nearly equal to that of the NaCl reference solution. These small increases in ^14,15^N_2_O_5_ and ^15,15^N_2_O_5_ suggest that either isotopic substitution of ^14,14^N_2_O_5_ with ^15^NO_3_^–^ does not occur efficiently or more likely that the isotopically labeled ^14,15^N_2_O_5_ and ^15,15^N_2_O_5_ products are lost through subsequent reactions prior to detection. The minor peaks at I­(^14,15^N_2_O_5_)^−^ and I­(^15,15^N_2_O_5_)^−^ observed when sampling from the reactor containing only NaCl solution are likely due to the natural abundance of isotopes in our N_2_O_5_ gas flow. The natural abundance of ^15^N (0.4%), ^17^O (0.04%), and ^18^O (0.2%) in our N_2_O_5_ source suggests that the peak height at 235.9 and 236.9 should be approximately 1% of that measured at 234.9. As shown in Figure 2A, the peak heights for I­(^14,15^N_2_O_5_)^−^ and I­(^15,15^N_2_O_5_)^−^ over the NaCl solution are consistent with these fractions.

**Figure 2:**
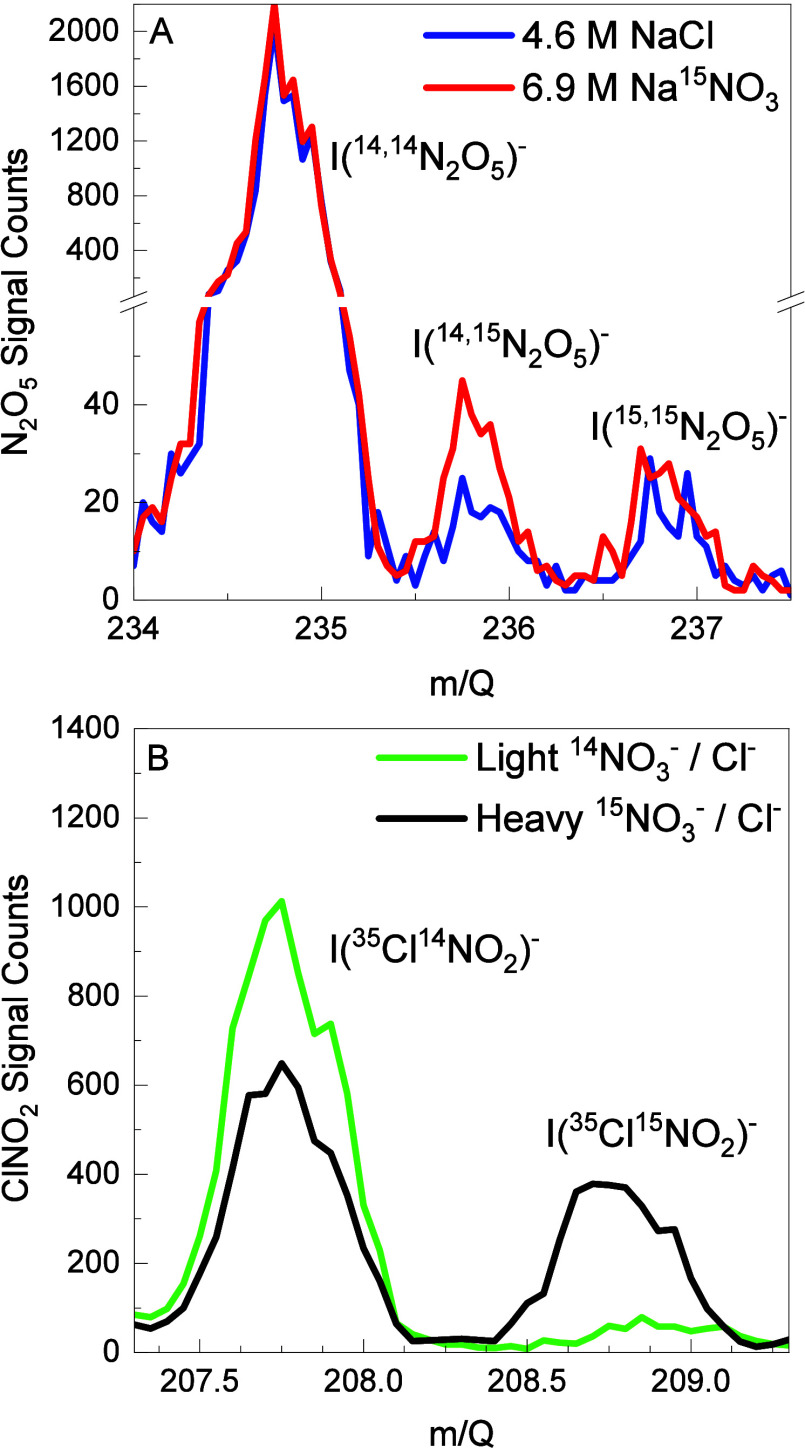
(A) Iodide CIMS mass spectra of the N_2_O_5_ species after flowing N_2_O_5_ over 6.9 M Na^15^NO_3_ in D_2_O (red) and 4.6 M NaCl in D_2_O (blue) (Panel A). (B) Mass spectra of the ClNO_2_ species after flowing N_2_O_5_ over mixed 3.7 M Na^14^NO_3_/0.02 M NaCl in D_2_O (green) and mixed 3.7 M Na^15^NO_3_/0.02 M NaCl in D_2_O (black). Note the small increase in ^14,15^N_2_O_5_ and even smaller change in ^15,15^N_2_O_5_ in (A) upon adding ^15^NO_3_^–^ in contrast to the large changes in ^14^ClNO_2_ and ^15^ClNO_2_ in (B).

### Dependence of ^15^ClNO_2_ Production on ^15^NO_3_^–^ and Cl^–^ Concentrations

Given the small concentrations of ^14,15^N_2_O_5_ and ^15,15^N_2_O_5_ at near-saturated Na^15^NO_3_ conditions (6.9 M Na^15^NO_3_ solution), we anticipated that extracting kinetic measurements from lower Na^15^NO_3_ concentrations would not be viable due to subsequent hydrolysis of the isotopically labeled nitration products in solution. We chose instead to add NaCl to solutions containing Na^15^NO_3_ in order to produce and measure Cl^14^NO_2_ and Cl^15^NO_2_. ClNO_2_ reacts slowly (*k*_ClNO_2_ + H_2_O_ = 270 s^–1^)^24^ and has low solubility in aqueous solution (*H* ≈ 0.02 M atm^–1^ in pure water),^35^ such that it evaporates before it can hydrolyze (as shown computationally in section 3.3 of ref 40). These features make this molecule both a “sink” for reacting N_2_O_5_ in solution and a “spy” for ^15^NO_3_^–^ reaction with ^14,14^N_2_O_5_ via the production of Cl^14^NO_2_ and Cl^15^NO_2_ ([Disp-formula eqR16] and [Disp-formula eqR17]). Figure 2B shows that the addition of NaCl to 3.7 M Na^15^NO_3_^–^ solutions containing labeled Na^15^NO_3_ results in the production of both Cl^14^NO_2_ and Cl^15^NO_2_. In contrast, the solution of NaCl and Na^14^NO_3_ only generates significant amounts of Cl^14^NO_2_. The presence of Cl^15^NO_2_ confirms that some N_2_O_5_ molecules undergo chemical exchange with ^15^NO_3_^–^ ions in solution before reacting with Cl^–^. The direct observation of Cl^15^NO_2_ production is common to all solutions investigated here and is a key result of our studies.

We next determine the relative rates of N_2_O_5_ reaction with nitrate and chloride by measuring the net fluxes of ^14,14^N_2_O_5_, ^14,15^N_2_O_5_, ^15,15^N_2_O_5_, Cl^14^NO_2_, and Cl^15^NO_2_ from solutions containing variable concentrations of Cl^–^ and ^15^NO_3_^–^. Figure 3 shows I^–^ CIMS signal intensities (proportional to concentration) for each of these species, measured at the mass-charge ratio associated with the iodide-adduct ion (e.g., I^–^(^14,14^N_2_O_5_)). The time series shown is for a 900 s observation window for ^15^NO_3_^–^ concentrations of 3.7 M ^15^NO_3_^–^ (Figure 3A,B) and 0.47 M ^15^NO_3_^–^ (Figure 3D,E). The time series alternate between N_2_O_5_ exposure to the NaCl reference solution (gray area) and the sample solution containing a mixture of Na^15^NO_3_ and NaCl (white area). Figure 3A shows that the singly exchanged I­(^14,15^N_2_O_5_)^−^ signal from the 3.7 M Na^15^NO_3_/0.02 M NaCl solution is clearly distinguishable from the signal over the saturated NaCl reference solution. The signal due to doubly exchanged I­(^15,15^N_2_O_5_)^−^ from the same solution is less distinct (Figure 3B). The I­(^14,15^N_2_O_5_)^−^ and I­(^15,15^N_2_O_5_)^−^ signals for the solution of 0.47 M Na^15^NO_3_/0.1 M NaCl (panels 3D and E) are significantly smaller than observed for the more concentrated nitrate solutions. Section II of the SI contains a statistical analysis of each N_2_O_5_ time series in Figure 3, which concludes that the differences in each panel are statistically significant at the 95% confidence level, though certainly weakest for panel E.

**Figure 3:**
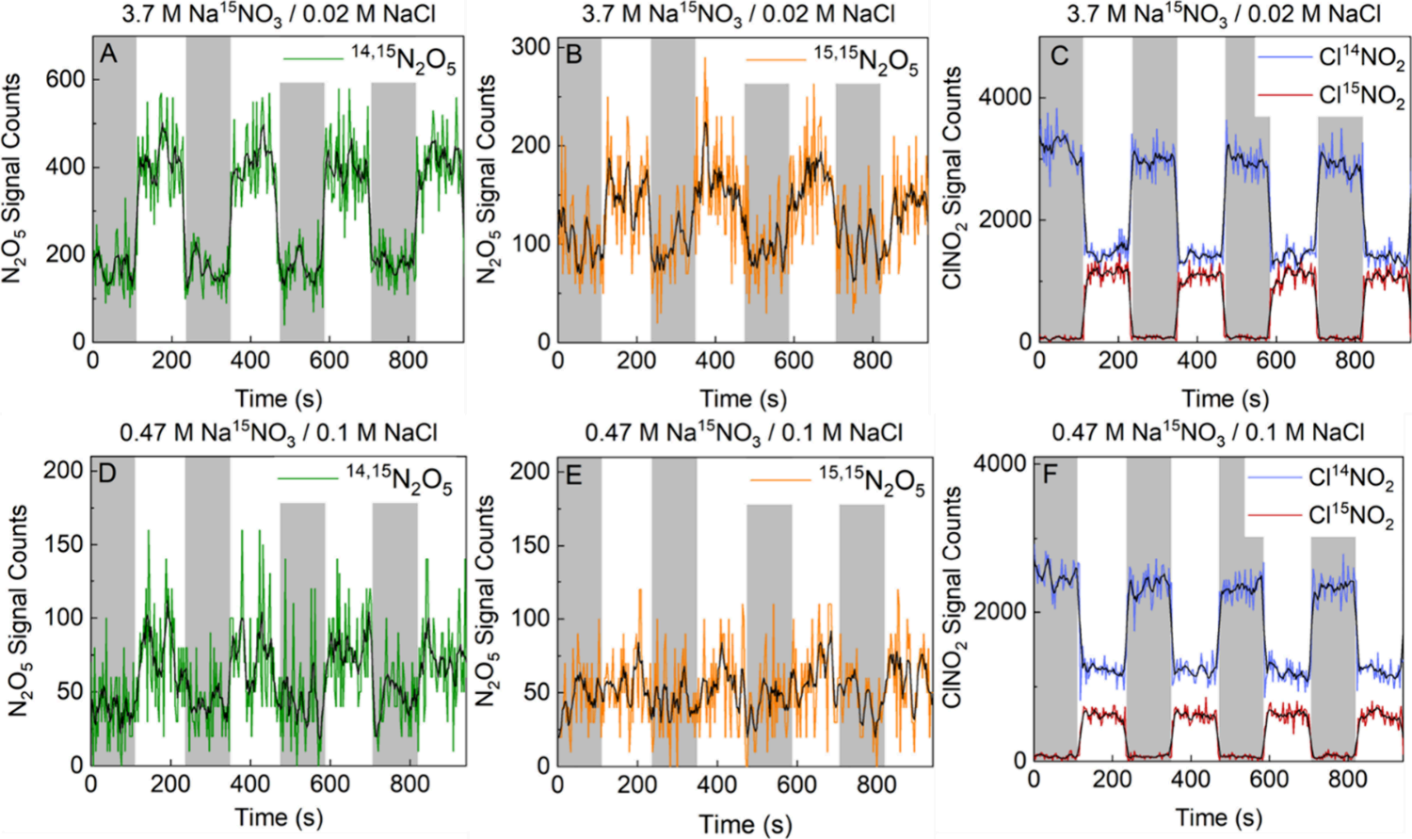
Isotopic exchange for 3.7 M ^15^NO_3_^–^ (Panels A–C) and 0.47 M ^15^NO_3_^–^ (Panels D–F). Top row shows the raw time series for ^14,15^N_2_O_5_ (A), ^15,15^N_2_O_5_ (B), and Cl^14^NO_2_ and Cl^15^NO_2_ (C) when N_2_O_5_ gas flow is alternated over saturated NaCl in D_2_O (gray area) and 3.7 M Na^15^NO_3_/0.02 M NaCl in D_2_O (white area). Bottom row (D–F) shows the same raw time series when N_2_O_5_ gas flow is alternated over saturated NaCl in D_2_O (gray area) and 0.47 M Na^15^NO_3_/0.1 M NaCl in D_2_O (white area). The solid black line is the 5-point moving average for each species.

While Figure 3 demonstrates that it is possible to track ^14,15^N_2_O_5_ and ^15,15^N_2_O_5_, particularly for 3.7 M NaNO_3_, these signals are much weaker than the Cl^14^NO_2_ and Cl^15^NO_2_ signals and barely visible for 0.47 M NaNO_3_. Here, we focus our analysis on the fraction of ^15^ClNO_2_ solely from the sample solution as this measurement does not rely on either the efficient transport of N_2_O_5_ through the experimental apparatus or the difference in ClNO_2_ production between the sample and reference boats, both of which can introduce error into the analysis.^41^ We therefore report ClNO_2_ measurements as a way of surveying the consequences of low and high NO_3_^–^ and Cl^–^ concentration on competitive NO_3_^–^ and Cl^–^ reactions.

Figure 3C,F show the time series for the Cl^14^NO_2_ and Cl^15^NO_2_ signals when N_2_O_5_ is alternated over the saturated NaCl reference solution (gray area) and the mixed NaCl/Na^15^NO_3_ solutions (white area). The sharp changes in the signal intensity reflect the fast response time of the system to switching the N_2_O_5_ flow between the solutions. The larger magnitude of signals from the ClNO_2_ species compared to the ^14,15^N_2_O_5_ and ^15,15^N_2_O_5_ species, as well as the greater difference in Cl^15^NO_2_ production between the reference and ^15^NO_3_^–^ solutions, reinforces our decision to measure the production of Cl^14^NO_2_ and Cl^15^NO_2_ as a proxy for the nitrate exchange rate, despite the statistically significant (but weak) production of ^14,15^N_2_O_5_ and ^15,15^N_2_O_5_.

We next use data like that in Panels 3C and 3F to construct the Cl^15^NO_2_ isotope fraction as the proportion of total ClNO_2_ produced that is Cl^15^NO_2_:

$${\mathrm{Cl}}^{15}{\mathrm{NO}}_{2}\;\mathrm{isotope}\;\mathrm{fraction}=\frac{\mathrm{Production}\;({\mathrm{Cl}}^{15}{\mathrm{NO}}_{2})}{\mathrm{Production}\;({\mathrm{Cl}}^{14}{\mathrm{NO}}_{2}\;\mathrm{and}\;{\mathrm{Cl}}^{15}{\mathrm{NO}}_{2})}=\frac{\mathrm{Signal}\;({\mathrm{Cl}}^{15}{\mathrm{NO}}_{2})}{\mathrm{Signal}\;({\mathrm{Cl}}^{14}{\mathrm{NO}}_{2})+\mathrm{Signal}\;({\mathrm{Cl}}^{15}{\mathrm{NO}}_{2})}$$ 1

We assume that evaporating Cl^14^NO_2_ and Cl^15^NO_2_ are detected with the same sensitivity in the I^–^ CIMS, and thus we can directly equate the recorded signal ratios to concentration ratios in the gas phase and thus to production rates in solution (eq 1). Figure 4A shows the Cl^15^NO_2_ isotope fraction plotted against [Cl^–^] at three different ^15^NO_3_^–^ concentrations. As can be seen, the Cl^15^NO_2_ isotope fraction increases with added Na^15^NO_3_ and decreases with added NaCl. Increasing ^15^NO_3_^–^ concentrations result in higher fractions of Cl^15^NO_2_ in the system, which would come from more N_2_O_5_ exchanging with ^15^NO_3_^–^ in solution to form ^14,15^N_2_O_5_ and ^15,15^N_2_O_5_ before the species undergo chlorination to form Cl^14^NO_2_ and Cl^15^NO_2_. Conversely, the presence of more Cl^–^ results in faster reactions of Cl^–^ with ^14,14^N_2_O_5_ and ^14,15^N_2_O_5_ before these species can undergo single or double ^15^NO_3_^–^ exchange.

**Figure 4:**
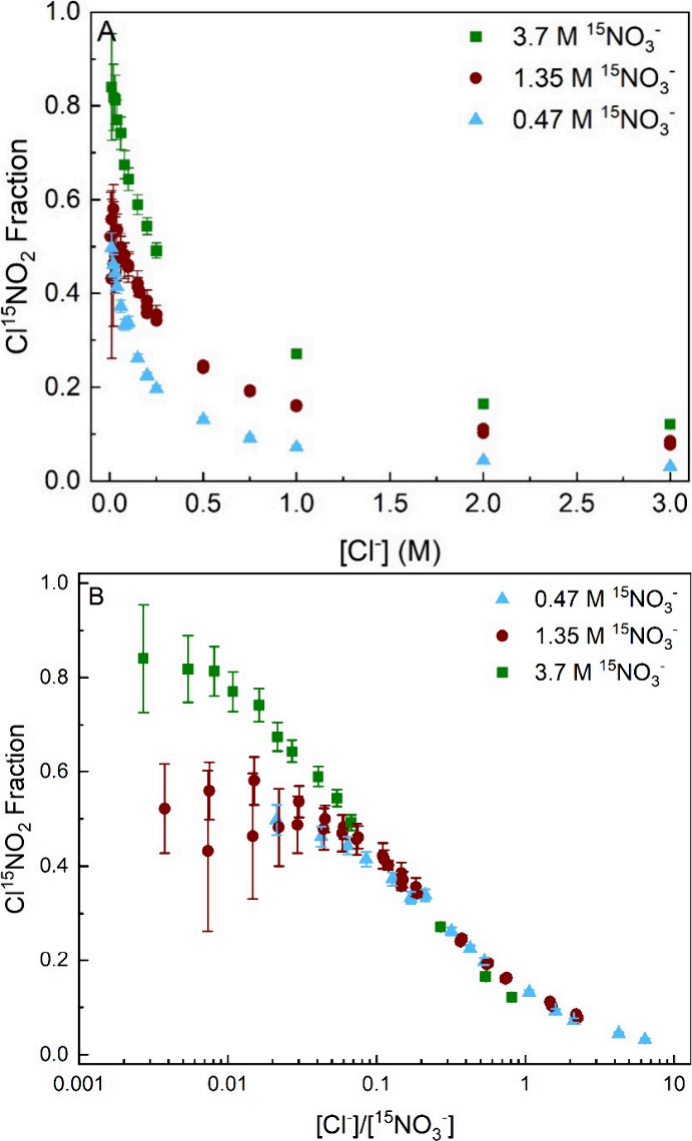
(A) Panel A shows the Cl^15^NO_2_ isotope fraction plotted against Cl^–^ concentration for ^15^NO_3_^–^ concentrations of 3.7, 1.35, and 0.47 M. The Cl^15^NO_2_ isotope fraction is defined as the Cl^15^NO_2_/(Cl^14^NO_2_ + Cl^15^NO_2_) signal ratio. The 1.35 M Na^15^NO_3_ data contain two separate runs of near equal concentration (1.33 and 1.36 M). Their overlap indicates good reproducibility in our experiments. The error bars are 95% confidence intervals, calculated from the propagated standard error of 120 data points each for Cl^14^NO_2_ and Cl^15^NO_2_. (B) Panel B presents the Cl^15^NO_2_ isotope fractions against [Cl^–^]/[^15^NO_3_^–^] for the different solutions. See Figure S5 of the SI (pages S15–17) for fits to the data in panel B using a steady-state solution of the full suite of S_N_2 rate equations.

All solutions were tested at high Cl^–^ concentrations up to or near saturation by adding up to 3.0 M NaCl to each Na^15^NO_3_ solution. The Cl^15^NO_2_ fraction at [Cl^–^] = 3.0 M and [^15^NO_3_^–^] = 3.7 M reaches 0.121 ± 0.005, indicating that even at nearly equal concentrations of Cl^–^ and ^15^NO_3_^–^, a significant amount of N_2_O_5_ still exchanges with ^15^NO_3_^–^ to form ^14,15^N_2_O_5_ and ^15,15^N_2_O_5_. The Cl^15^NO_2_ fraction at [Cl^–^] = 3.0 M and [^15^NO_3_^–^] = 0.47 M reaches 0.031 ± 0.003, revealing isotopically labeled N_2_O_5_ is still created in solution despite the high [Cl^–^]/[^15^NO_3_^–^] ratio of 6:1. Panel A further reveals when NO_3_^–^ exchange is expected before chlorination by identifying where the data crosses 0.5 isotope exchange. In the 3.7 M ^15^NO_3_^–^ solution, Cl^–^ will more often react with a nitrate-exchanged N_2_O_5_ to produce Cl^15^NO_2_ at Cl^–^ concentrations up to 0.25 M NaCl. In the case of 0.47 M ^15^NO_3_^–^, the crossing point occurs at only 0.01 M NaCl. See Section V of the SI for analytic expressions for the fraction of each isotopic species in solution.

It should be noted that, if Cl^14^NO_2_ was formed exclusively from ^14,15^N_2_O_5_, the maximum Cl^15^NO_2_ fraction would be 0.5 if we assume that every initial ^14,14^N_2_O_5_ undergoes exchange to form ^14,15^N_2_O_5_ prior to chlorination. This 0.5 limit is a result of [Disp-formula eqR16], where ^14,15^N_2_O_5_* + Cl^–^ → 12 Cl^14^NO_2_ + 12 ^15^NO_3_^–^ + 12 Cl^15^NO_2_ + 12 ^14^NO_3_^–^ has an equal probability of forming either ClNO_2_ isotope. However, Figure 4A demonstrates that the Cl^15^NO_2_ fraction rises above 0.5 for the 1.35 and 3.7 M ^15^NO_3_^–^ solutions, even reaching 0.8 ± 0.1 (95% confidence interval) in 3.7 M ^15^NO_3_^–^. For the Cl^15^NO_2_ isotope fraction to exceed 0.5, there must be some fraction of Cl^15^NO_2_ formed from the chlorination of ^15,15^N_2_O_5_. In particular, a Cl^15^NO_2_ isotope fraction of 0.8 implies that 80% of the N atoms in N_2_O_5_ are ^15^N. We derive the fractions of each N_2_O_5_ isotope in Section V of the SI and find that an 80% Cl^15^NO_2_ isotope fraction corresponds to ^15,15^N_2_O_5_, ^14,15^N_2_O_5_, and ^14,14^N_2_O_5_ mole percentages of 71, 18, and 11% respectively, implying significant N_2_O_5_ double exchange.

The Cl^15^NO_2_ isotope fraction in Figure 4A is alternately plotted on a log scale against the concentration ratio [Cl^–^]/[NO_3_^–^] in panel B. This panel highlights the decrease in the Cl^15^NO_2_ fraction with increasing [Cl^–^]/[NO_3_^–^]. It also demonstrates that the data sets converge at high [Cl^–^]/[NO_3_^–^] while differing at lower values. As described in Section IV of the SI, the convergence at high [Cl^–^]/[NO_3_^–^] reflects the competition between nitration and chlorination, which in turn controls Cl^15^NO_2_ production. At low [Cl^–^]/[NO_3_^–^], the differing isotope fractions instead reflect the competition between hydrolysis and nitration. In this limit, faster nitration in 3.7 M ^15^NO_3_^–^ than in 1.35 M ^15^NO_3_^–^ means that hydrolysis is slower in removing ^14,15^N_2_O_5_ and ^15,15^N_2_O_5_ that feed Cl^15^NO_2_ production, and therefore that the Cl^15^NO_2_ isotope fraction rises. Figure S5 of the SI shows quantitative steady-state fits to the data in Figure 4B using rate constants reported below and the S_N_2 mechanism described by [Disp-formula eqR6]–[Disp-formula eqR11].

### Determination of *k*_Cl^–^_ */* *k*_NO_3_^–^_ from Measurement-Model Comparisons of Cl^15^NO_2_

A primary objective of this study is the determination of the nitration rate constant *k*_NO_3_^–^_ relative to the chlorination rate constant *k*_Cl^–^_ ([Disp-formula eqR10] and [Disp-formula eqR11]). We can extract *k*_Cl^–^_ */k*_NO_3_^–^_ by constraining the KM model with our observations of the Cl^15^NO_2_ isotope fractions. The KM model employs single values of rate constants and diffusion coefficients throughout solution and does not take into account molecularly resolved interfacial structure or interfacial partitioning and reaction.^44,45^ The parameters listed in Tables S1 and S2 of the SI yield a N_2_O_5_ bulk-phase reacto-diffusive length^13^ (*D*_b_/*k*)^1/2^ of approximately 70 nm using a N_2_O_5_ bulk diffusion constant *D*_b_ = 1.9 × 10^–5^ cm^2^ s^–1^ and reaction rate constant *k* = 4.0 × 10^5^ s^–1^ for activation of N_2_O_5_ to N_2_O_5_*. This depth spans 15 distinct layers of the KM model. We adjust the rate constant ratio *k*_Cl^–^_ */k*_NO_3_^–^_ in the model to best match the measured Cl^15^NO_2_ isotope fraction, as shown in Figure 5.

**Figure 5:**
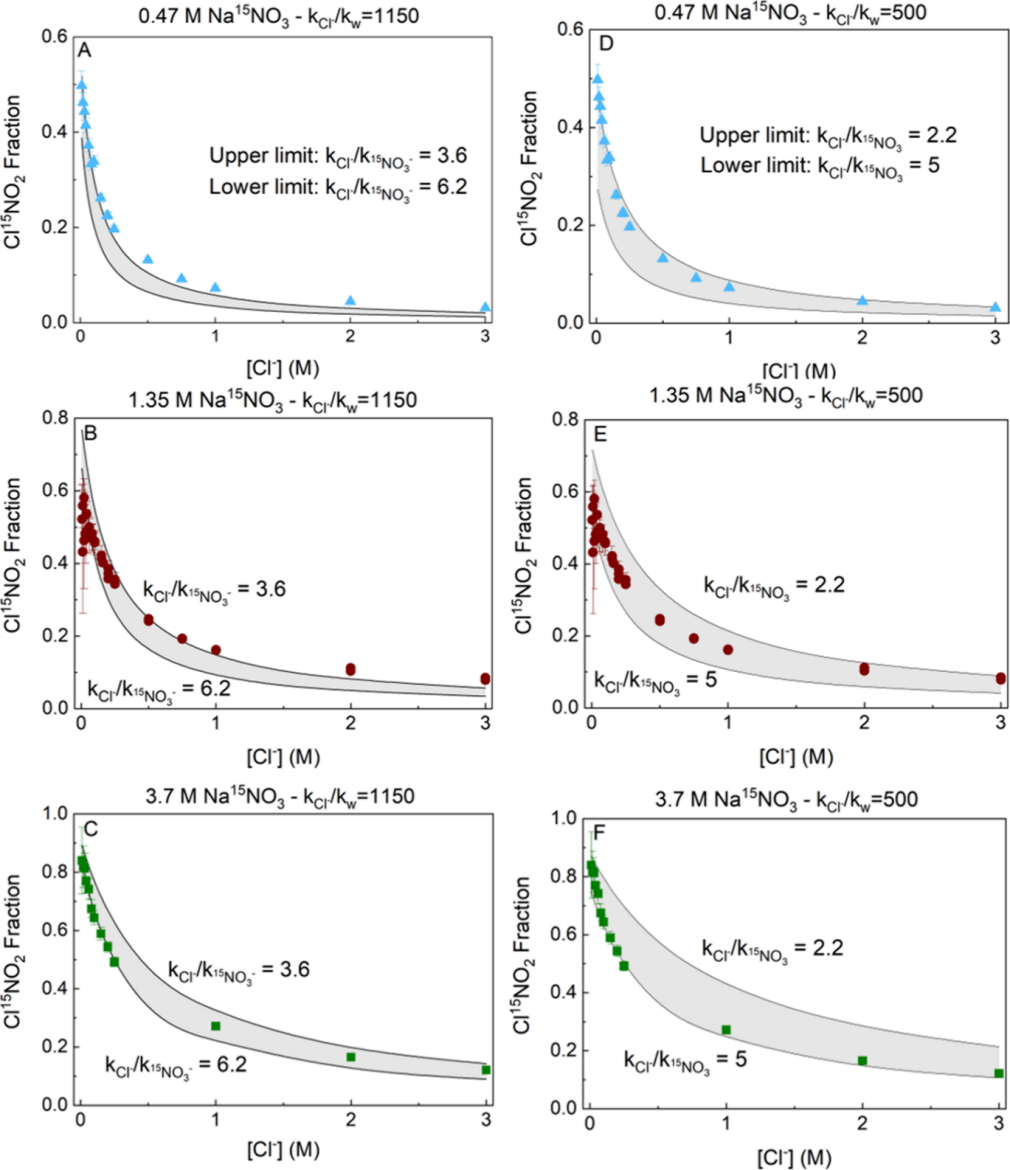
(Panels A–C) Modeled Cl^15^NO_2_ fraction with *k*_Cl^–^_ */k*_NO_3_^–^_ = 3.6 (upper limit) and 6.2 (lower limit) for *k*_Cl^–^_ */k*_w_ = 1150. (Panels D–F) Modeled Cl^15^NO_2_ fraction with *k*_Cl^–^_ */k*_NO_3_^–^_ = 2.2 (upper limit) and 5 (lower limit) for *k*_Cl^–^_ */k*_w_ = 500. Experimental measurements of the Cl^15^NO_2_ fraction are included with colored symbols on each figure.

The kinetic model employs a simplified reaction mechanism because we lack knowledge about the nature or location of the N_2_O_5_* deactivation steps [Disp-formula eqR-8] and [Disp-formula eqR11]. The exact values of these deactivation rates and even their presence are not necessary to determine the *k*_Cl^–^_ */k*_NO_3_^–^_ ratio as long as N_2_O_5_* is the same reactant species for hydrolysis, chlorination, and nitration. We also assume that NO_3_^–^ chemical exchange deactivates reactant N_2_O_5_* to produce inactivated N_2_O_5_. As described in Section VII of the SI, these assumptions mean that the S_N_1 and S_N_2 mechanisms become equivalent and NO_2_^+^ and N_2_O_5_* are interconvertible species in the reaction steps. This simplification further enables a detailed comparison with prior studies of the nitrate effect presented later.

The modeled and measured Cl^15^NO_2_ isotope fractions are brought into agreement in Figure 5 using *k*_Cl^–^_ */k*_NO_3_^–^_ rate constant ratios between 2 and 6 for all combinations of ^15^NO_3_^–^ and Cl^–^ concentrations. These measurements include two Cl^–^ concentration limits for the *k*_Cl^–^_ */k*_w_ rate constant ratio (1150 (left panels) and 500 (right panels). We first focus on the dependence of *k*_Cl^–^_ */k*_NO_3_^–^_ on nitrate concentration using *k*_Cl^–^_ */k*_w_ = 1150, the value determined by Kregel et al. for [Cl^–^] between 0.0054 and 0.21 M, representative of the lowermost chloride concentrations used here (Figure 5A–C). For the lowest Na^15^NO_3_ concentration (0.47 M, Figure 5A) the measurements are best fit by a model with *k*_Cl^–^_ */k*_NO_3_^–^_ = 3.6, whereas the highest Na^15^NO_3_ concentration (3.7 M, Figure 5C) the measurements are best fit by a model with *k*_Cl^–^_ */k*_NO_3_^–^_ = 6.2. This is particularly pronounced at low chloride concentration (Cl^–^ < 0.5 M).

We also explore the impact of a lower value of *k*_Cl^–^_ */k*_w_ = 500 on our retrieved *k*_Cl^–^_ */k*_NO_3_^–^_ ratio. Kregel et al. found that the *k*_Cl^–^_ */k*_w_ ratio decreases from 1240 ± 90 to 840 ± 50 between [Cl^–^] = 0.0054 and 0.21 M, and then drops with significant uncertainty to 500 ± 500 at measurements of 0.5 and 2.4 M Cl^–^ (error bars represent 95% confidence intervals).^41^ As shown in Figure 5D–F, our observations of the Cl^15^NO_2_ fraction are consistent with a *k*_Cl^–^_ */k*_NO_3_^–^_ ranging between 2.2 and 5.0 for this lower *k*_Cl^–^_ */k*_w_ ratio, as compared with 3.6–6.2 for *k*_Cl^–^_ */k*_w_ = 1150. The values of *k*_Cl^–^_ */k*_NO_3_^–^_ therefore overlap across the two ranges between ∼3.5 and ∼5.

Figure 5 shows the fitted *k*_Cl^–^_ */k*_NO_3_^–^_ ratio is larger for the higher NaNO_3_ concentrations. This is most evident in the low Cl^–^ regime, where *k*_Cl^–^_ */k*_NO_3_^–^_ is best fit at *k*_Cl^–^_ */k*_NO_3_^–^_ = 3.6 (Panel A) or 2.2 (Panel D) for 0.47 M NaNO_3_ and is best fit for *k*_Cl^–^_ */k*_NO_3_^–^_ = 6.2 (Panel C) or 5.0 (Panel F) for 3.7 M NaNO_3_. The fitted *k*_Cl^–^_ */k*_NO_3_^–^_ ratio is also larger at lower NaCl concentrations at fixed NaNO_3_ concentration. This trend can be seen by the underfitting of the data at [NaCl] > 1 M in most panels, such that a lower *k*_Cl^–^_ */k*_NO_3_^–^_ ratio would be required to fit the data.

One potential explanation could be an increase in the interfacial concentration of NO_3_^–^ with respect to the bulk at increasing NaCl concentrations. Wingen et al. present MD simulations of the density profiles of ions in concentrated NaCl and NaNO_3_ solutions. They suggest that Cl^–^ ions near the surface attract Na^+^ ions, which in turn pull NO_3_^–^ ions toward the surface through formation of coupled Cl^–^/Na^+^ and Na^+^/NO_3_^–^ double layers.^46^ Enhanced interfacial concentrations of ^15^NO_3_^–^ could in turn lead to increased exchange to form ^14,15^N_2_O_5_ and ^15,15^N_2_O_5_ not accounted for in our kinetic model. MD simulations of concentrated NaNO_3_ with no halide solutions found little interfacial apportionment of NO_3_^–^,^46,47^ suggesting that added Cl^–^ aids NO_3_^–^ exchange with N_2_O_5_. This effect may help to explain the lack of ^15^NO_3_^–^ exchange detected using 6.9 M Na^15^NO_3_ solution without added Cl^–^ in Figure 2A, where only a small peak of ^14,15^N_2_O_5_ was present with no noticeable ^15,15^N_2_O_5_. It is thus possible that added NaCl draws more NO_3_^–^ to the surface through correlated Cl^–^/Na^+^/NO_3_^–^ attractions.

### Production of Cl^15^NO_2_ from Single and Multiple Collisions of ^14,15^N_2_O_5_ and ^15,15^N_2_O_5_

From our measurements alone, it is unclear whether Cl^15^NO_2_ is formed during a single encounter of ^14,14^N_2_O_5_ with the solution (i.e.,^14,14^N_2_O_5_ is converted to Cl^15^NO_2_ before the evaporation of ^14,15^N_2_O_5_ and/or ^15,15^N_2_O_5_) or if Cl^15^NO_2_ production relies on multiple entry and evaporation steps of ^14,15^N_2_O_5_ and^15,15^N_2_O_5_ to produce Cl^15^NO_2_. To address this question, we run the model under a configuration where ^14,15^N_2_O_5_ and ^15,15^N_2_O_5_ that evaporate from the solution are not permitted to reenter solution. In this configuration the only pathway to Cl^15^NO_2_ production is from the adsorption of ^14,14^N_2_O_5_ and subsequent nitration prior to chlorination. The results of the two simulations are shown in Figure 6, where the base case model result (^14,14^N_2_O_5_, ^14,15^N_2_O_5_ and ^15,15^N_2_O_5_ are all permitted to enter and react) is shown in the black line and the model where only ^14,14^N_2_O_5_ adsorption is permitted to enter is shown in the red line. The difference between the black and red lines represents Cl^15^NO_2_ produced from ^14,15^N_2_O_5_ and ^15,15^N_2_O_5_ that exited solution and then reenter and react with Cl^–^. The modeled Cl^15^NO_2_ fraction decreases by 18% at 0.01 M Cl^–^ and 45% at 3.0 M Cl^–^ when the adsorption of ^14,15^N_2_O_5_ and ^15,15^N_2_O_5_ is turned off. This comparison indicates that a majority of Cl^15^NO_2_ formed does not come from a ^14,15^N_2_O_5_ or ^15,15^N_2_O_5_ molecule leaving solution and then reentering to react with Cl^–^. Instead, most Cl^15^NO_2_ are produced when ^14,14^N_2_O_5_ molecules enter solution and undergo single or double ^15^NO_3_^–^ exchange before chlorination.

**Figure 6:**
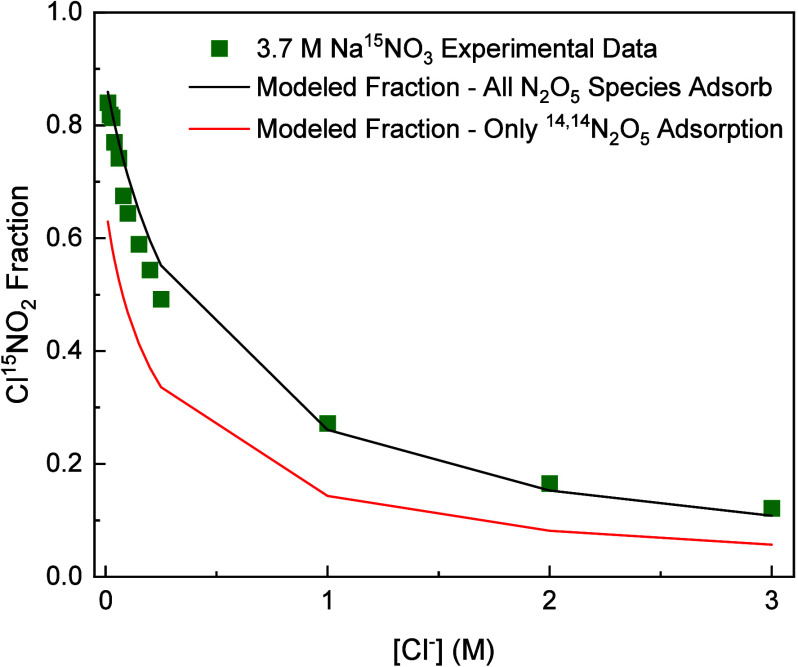
Dependence of the Cl^15^NO_2_ fraction on chloride (3.7 M Na^15^NO_3_) for two model scenarios, using rate constant ratios of *k*_Cl^–^_ */k*_w_ = 1150 and *k*_Cl^–^_ */k*_NO_3_^–^_ = 5. The base case model result (where ^14,14^N_2_O_5_, ^14,15^N_2_O_5_ and ^15,15^N_2_O_5_ are all permitted to adsorb and react) is shown with the black line, and the model where only ^14,14^N_2_O_5_ adsorption is permitted is shown in the red line.

### Potential Cl^15^NO_2_ Production from the Reaction of Cl^14^NO_2_ with ^15^NO_3_^–^

We also explored the possibility that Cl^15^NO_2_ could be formed through a competing pathway not involving ^14,15^N_2_O_5_ or ^15,15^N_2_O_5_, that is the direct reaction of Cl^14^NO_2_ with ^15^NO_3_^–^ to form Cl^15^NO_2_ and ^14^NO_3_^–^. We can gauge whether this reaction contributes to Cl^15^NO_2_ production by passing Cl^14^NO_2_ (in the absence of ^14,14^N_2_O_5_) over a concentrated Na^15^NO_3_ solution and monitoring Cl^15^NO_2_ production. In this test, N_2_O_5_ was first bubbled through a 5 M NaCl solution, which quantitatively converts ^14,14^N_2_O_5_ to Cl^14^NO_2_. No N_2_O_5_ was detected in the outflow by the I^–^ CIMS, indicating that all N_2_O_5_ reacts in solution. The resulting Cl^14^NO_2_ gas was then alternately flowed over saturated, 7 M Na^15^NO_3_ or pure D_2_O. We detected no difference in the Cl^15^NO_2_ signal between the two solutions, with a signal intensity of Cl^15^NO_2_ over the Na^15^NO_3_ solution that was only about 1% of the measured Cl^14^NO_2_ from the bubbler. These results indicate that reaction of gaseous Cl^14^NO_2_ with a ^15^NO_3_^–^ solution is too slow to produce Cl^15^NO_2_ in our setup. This test is not a precise mimic of our experiments, however, which instead generate Cl^14^NO_2_ by chlorination of dissolved ^14,14^N_2_O_5_. The 100-fold higher solubility^24,35^ of N_2_O_5_ implies that ClNO_2_ is produced deeper in solution, providing more time for Cl^14^NO_2_ to potentially react with ^15^NO_3_^–^ before it evaporates. Future studies, perhaps involving the merging of saturated droplets of Cl^14^NO_2_ and Na^15^NO_3_, may help to reveal the time scale and extent of nitrate exchange of ClNO_2_ in solution.

### Dependence of the ClNO_2_ Product Yield on Nitrate Concentration

Bertram and Thornton showed that the addition of Cl^–^ to NO_3_^–^ containing aerosol particles can overcome the nitrate effect. In these experiments, the reactive uptake coefficient returned to ∼0.03 for a Cl^–^/NO_3_^–^ mole ratio > 0.2,^17^ although the results here indicate exchange of ^15^NO_3_^–^ with N_2_O_5_ still occurs at this mole ratio. To investigate the competition between NO_3_^–^ and Cl^–^ reactions with N_2_O_5_ as well as any potential enhancement of Cl^–^ reactivity with ^14^NO_3_^–^ present, we measured the product yield of Cl^14^NO_2_ at three different Cl^–^ concentrations (Figure 7). We use the same definition for product yield as Kregel et al:^41^

$${\mathrm{ClNO}}_{2}\;\mathrm{Product}\;\mathrm{Yield}=\frac{\mathrm{ClN}O_{2}\;\mathrm{production}}{N_{2}O_{5}\;\mathrm{reaction}}=\frac{\mathrm{Signal}\;\mathrm{ClN}{O_{2}}_{\mathrm{Sample}}}{\mathrm{Signal}\;\mathrm{ClN}{O_{2}}_{\mathrm{Reference}}}$$ 2

**Figure 7:**
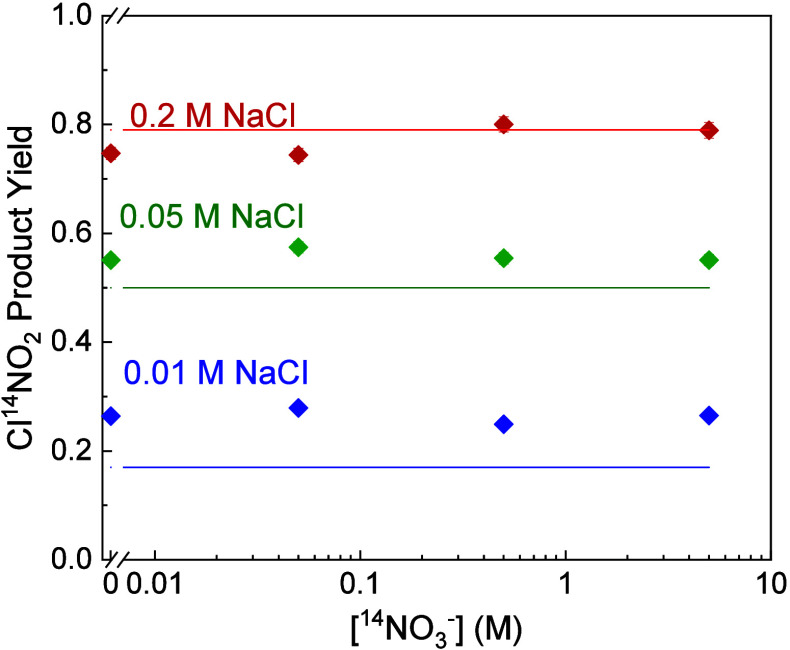
Dependence of the Cl^14^NO_2_ product yield on [^14^NO_3_^–^] (0, 0.05, 0.5, and 5 M) at three different Cl^–^ concentrations (0.01, 0.05, and 0.2 M). The solid lines are the ClNO_2_ product yields predicted from our model for these 3 Cl^–^ concentrations. The modeled product yield is calculated from the net ClNO_2_ desorption flux and the net N_2_O_5_ adsorption flux. A single *k*_Cl^–^_ */k*_w_ value of 1150 is used for all model calculations.

The reference solution, saturated NaCl in D_2_O, corresponds to complete conversion of reacted N_2_O_5_ to ClNO_2_,^11,41^ and thus the product yield is a measure of the competition between chlorination and hydrolysis of N_2_O_5_, as well as any differential effects of added NO_3_^–^ on these reaction rates. The Cl^14^NO_2_ product yield in Figure 7 does not change with the addition of NO_3_^–^, even at 5 M NO_3_^–^, indicating that the presence of NO_3_^–^ and its exchange with N_2_O_5_ do not alter the relative chlorination and hydrolysis rates. This null effect contrasts with the effect of other ions on ClNO_2_ production. In particular, the presence of acetate and sulfate has been shown to decrease the fraction of ClNO_2_ produced, suggesting that these ions do compete with Cl^–^ for N_2_O_5_. In the Discussion, we expand on potential reasons for these differences.

## Discussion

The direct detection of isotopically labeled ^14,15^N_2_O_5_, ^15,15^N_2_O_5_, and Cl^15^NO_2_ confirms that chemical exchange can occur between ^14,14^N_2_O_5_ and ^15^NO_3_^–^ in solution prior to evaporation of N_2_O_5_ or its reaction with Cl^–^ or D_2_O. Nitration prior to evaporation was inferred earlier by Gržinić et al. through measurements of the greater reactive uptake of labeled ^13,14^N_2_O_5_ than of ^14,14^N_2_O_5_.^25^ In the absence of isotopic labeling, this NO_3_^–^ exchange with N_2_O_5_ becomes a “hidden” pathway. We compare below the rate of NO_3_^–^ attack on N_2_O_5_ with attack by H_2_O, Cl^–^, SO_4_^2–^, and CH_3_COO^–^ and explore different mechanisms for these reactions. We then analyze the implications of the newly determined *k*_NO_3_^–^_ */k*_w_ rate constant ratio on parameterizations of N_2_O_5_ reactive uptake and compare it with earlier measurements of the nitrate effect.

Table 1 combines data from ClNO_2_ product yield measurements and kinetic analysis reported here for nitration and chlorination with measurements of hydrolysis and chlorination by Kregel et al.^41^ The two regimes in Table 1 correspond to NaCl concentrations from 0.005 to 0.2 M and from 0.54 to 2.4 M, over which a steadily decreasing ratio of *k*_Cl^–^_ */k*_w_ with increasing NaCl concentration was measured. The study here follows a similar range in [Cl^–^] from 0.005 to 3.0 M. The reaction rate constant ratios involving *k*_Cl^–^_ (chlorination), *k*_NO_3_^–^_ (nitration), and *k*_w_ (hydrolysis) of N_2_O_5_ follow the order *k*_Cl^–^_ > *k*_NO_3_^–^_ ≫ *k*_w_, such that chlorination and nitration are each much faster than hydrolysis. Despite the large *k*_Cl^–^_ */k*_w_ ratio of 500 to 1000, temperature studies by Kregel et al. indicate that the activation energy is only 3.0 ± 1.5 kJ/mol lower for chlorination than for hydrolysis (equal to 1.2 *RT*).^41^ The high *k*_Cl^–^_ */k*_w_ ratio is instead attributed to an increase in entropy as tightly bound water molecules around Cl^–^ relax as the Cl-NO_2_ bond is formed. Given the nearly equal hydration entropies of Cl^–^ (−97 J/mol/K) and NO_3_^–^ (−99 J/mol/K),^48^ it is intriguing to speculate that NO_3_^–^ follows a similar pathway, but perhaps with a slightly higher activation energy as reflected by its lower rate constant.

**Table 1:** Rate Constant Ratios at 25°C for Chlorination, Hydrolysis, and Nitration of N_2_O_5_ in D_2_O and H_2_O (reactions R 9, 10, 11)

rate constant ratio	*k*_Cl^–^_ */k*_D_2_O_ at lower [NaCl]	*k*_Cl^–^_ */k*_D_2_O_ at higher [NaCl]
*k*_Cl^–^_ */k*_D_2_O_	1150 ± 90^a^	500^b^
*k*_Cl^–^_ */k*_NO_3_^–^_ ^c^	3.6–6.2	2.2–5.0
*k*_NO_3_^–^_ */k*_D_2_O_	190–320	100–230
*k*_NO_3_^–^_ */k*_H_2_O_ ^d^	130–230	70–160

a *k*_Cl^–^_ */k*_D_2_O_ value of 1150 ± 90 comes from the average of *k*_Cl^–^_ */k*_D_2_O_ of solutions between 0.0054 and 0.21 M NaCl in Kregel et al.^41^

b *k*_Cl^–^_ */k*_D_2_O_ value of 500 comes from the *k*_Cl^–^_ */k*_D_2_O_ of 0.54 and 2.4 M NaCl solutions in Kregel et al.^41^ These values are 500 ± 400 and 500 ± 600, respectively.

c See Section X of the SI for changes in the ratio constant ratio when NaCl and NaNO_3_ activity coefficients are included.

d All experiments were carried out in D_2_O. To determine the *k*_NO_3_^–^_ */k*_H_2_O_ ratio, we reduce the *k*_NO_3_^–^_ */k*_*D*_2_ *O*_ ratio by a factor of 1.4. This factor comes from Kregel et al., who found that *k*_Cl^–^_ */k*_D_2_O_ is approximately 1.4 times larger than *k*_Cl^–^_ */k*_H_2_O_ at 5 °C.^41^

With *k*_Cl^–^_ */k*_w_ from Kregel et al.^41^ and *k*_Cl^–^_ */k*_NO_3_^–^_ from the KM model used in this study, we can derive *k*_NO_3_^–^_ */k*_w_ ratios of 190–320 and 100–230 for the two regimes in Table 1. As both studies utilized D_2_O as the solvent, we refer to this ratio as *k*_NO_3_^–^_ */k*_D_2_O_. Additionally, Kregel et al. found that a change in solvent from D_2_O to H_2_O reduced *k*_Cl^–^_ */k*_w_ by a factor of roughly 1.4,^41^ such that *k*_NO_3_^–^_ */k*_D_2_O_ might be reduced by a similar magnitude and yield *k*_NO_3_^–^_ */k*_H_2_O_ ratios of 130–230 and 70–160. These H_2_O values are also listed in Table 1.

The ratios in Table 1 can be extended to reactions of other ions by combining data from previous studies of the suppression of the ClNO_2_ product yield by added sulfate and acetate.^40,41,49^ The lower observed production of ClNO_2_ found with added SO_4_^2–^ or CH_3_COO^–^ in solution contrasts with the lack of an effect by added NO_3_^–^ in Figure 7. This stark difference likely arises from the distinct ways in which N_2_O_5_ reacts with these ions. Karimova and Gerber postulate that sulfate and acetate undergo S_N_2 reactions with N_2_O_5_ via transition states (in braces) listed below that yield reactive intermediates (in brackets),^29,40^ along with a parallel reaction for NO_3_^–^:

$${{\mathrm{SO}}_{4}}^{2-}+N_{2}{O_{5}}^{*}\to {\left\{{\mathrm{SO}}_{4}{\mathrm{NO}}_{2}{\mathrm{NO}}_{3}\right\}}^{2-}\to {\left[{\mathrm{SO}}_{4}{\mathrm{NO}}_{2}\right]}^{-}+{{\mathrm{NO}}_{3}}^{-}$$

$${\mathrm{CH}}_{3}{\mathrm{COO}}^{-}+N_{2}{O_{5}}^{*}\to {\left\{{\mathrm{CH}}_{3}{\mathrm{COONO}}_{2}{\mathrm{NO}}_{3}\right\}}^{-}\to \left[{\mathrm{CH}}_{3}{\mathrm{COONO}}_{2}\right]+{{\mathrm{NO}}_{3}}^{-}$$

$${{\mathrm{NO}}_{3}}^{-}+N_{2}{O_{5}}^{*}\to {\left\{{\mathrm{NO}}_{3}{\mathrm{NO}}_{2}{\mathrm{NO}}_{3}\right\}}^{-}\to \left[N_{2}O_{5}\right]+{{\mathrm{NO}}_{3}}^{-}$$

In the case of sulfate and acetate, the intermediates [SO_4_NO_2_]^−^ and [CH_3_COONO_2_] quickly react with surrounding water molecules to produce NO_3_^–^ and H^+^. They also regenerate the original SO_4_^2–^ and CH_3_COO^–^ ions, which essentially act as catalysts that destroy N_2_O_5_.^29^ For NO_3_^–^ attack on N_2_O_5_, the analogous reaction intermediate is just N_2_O_5_ itself. Added nitrate therefore has no effect on the branching between hydrolysis and chlorination, even as it suppresses the overall reaction rate by deactivating N_2_O_5_* (or reacting with NO_2_^+^ in the S_N_1 mechanism).

We can use the ClNO_2_ product yields in the presence of sulfate, acetate, and nitrate to estimate relative rate constants for attack by each of these ions. At equal 0.5 M bulk concentrations of Cl^–^ and SO_4_^2–^, and Cl^–^ and CH_3_COO^–^, Staudt et al. found that the product yield of ClNO_2_ decreases from 0.80 with just Cl^–^ to 0.47 with added SO_4_^2–^ and to 0.18 with added CH_3_COO^–^.^40^ These product yields may be represented as rate constant ratios:

$${\mathrm{ClNO}}_{2}\;\mathrm{product}\;\mathrm{yield}\;\left(\mathrm{sulfate}\right)=\frac{1}{\frac{k_{w}\left[H_{2}O\right]}{k_{{\mathrm{Cl}}^{-}}\left[{\mathrm{Cl}}^{-}\right]}+\frac{k_{{\mathrm{SO}}_{4}^{2-}}\left[{\mathrm{SO}}_{4}^{2-}\right]}{k_{{\mathrm{Cl}}^{-}}\left[{\mathrm{Cl}}^{-}\right]}+1}=0.47$$

and

$${\mathrm{ClNO}}_{2}\;\mathrm{product}\;\mathrm{yield}\;(\mathrm{acetate})=\frac{1}{\frac{k_{w}[H_{2}O]}{k_{{\mathrm{Cl}}^{-}}[{\mathrm{Cl}}^{-}]}+\frac{k_{{\mathrm{CH}}_{3}{\mathrm{COO}}^{-}}[{\mathrm{CH}}_{3}{\mathrm{COO}}^{-}]}{k_{\mathrm{Cl}-}[{\mathrm{Cl}}^{-}]}+1}=0.18$$

The Staudt et al. value of *k*_Cl^–^_ */k*_w_ = 440 with [H_2_O] = 55 M yields *k*_Cl^–^_ */k*_SO_4_^2–^_ ≈ 1.1 and *k*_Cl^–^_ */k*_CH_3_COO^–^_ ≈ 0.2. Connecting these rate constant ratios to *k*_NO_3_^–^_ via *k*_Cl^–^_ */k*_NO_3_^–^_ = 2–6, we obtain *k*_SO_4_^2–^_/*k*_NO_3_^–^_ ≈ 1.9–5 and *k*_CH_3_COO^–^_/*k*_NO_3_^–^_ ≈ 9–30 over the range of values in Table 1. At 0.5 M ions, this indicates that sulfate and acetate are more reactive with N_2_O_5_ than NO_3_^–^ is. We note, however, that sulfate, acetate, and chloride likely distribute themselves differently in the interfacial region where N_2_O_5_ reactions may preferentially occur,^46,50–52^ so that these rate constant ratios should be considered effective ones with respect to bulk-phase ion concentrations. In particular, molecular dynamics simulations indicate sulfate is repelled from the surface^51^ while acetate is weakly redistributed,^52^ and as noted above, Cl^–^ may draw NO_3_^–^ to the surface.^46^ The importance of both Cl- and N_2_O_5_ interfacial distributions for Cl^–^ attack on N_2_O_5_ has been emphasized by Moon and Limmer,^35^ who predict that this S_N_2 reaction is enhanced through formation of a Cl^–^/N_2_O_5_ ion-dipole complex that is less solvated in the interfacial region, followed by the stabilization of a charged-delocalized transition state that mimics the surface propensity of a large anion.

### Comparisons with Reactive N_2_O_5_ Uptake into NaNO_3_ Solutions

Prior reactive uptake measurements by Bertram and Thornton, using an entrained aerosol flow reactor, yield a *k*_Cl^–^_ */k*_NO_3_^–^_ ratio of 29 ± 6.^17^ This value surpasses our range of 2–6 in Table 1 extracted from the Cl^15^NO_2_ isotope fractions. Although we have not measured N_2_O_5_ reactive uptake in these studies, we can use our derived *k*_NO_3_^–^_ */k*_H_2_O_ ratios in Table 1 to predict the impact of dissolved NO_3_^–^ on N_2_O_5_ reactive uptake coefficient γ in H_2_O solutions. Figure 8A shows the effect of 0 to 7 M NaNO_3_ on N_2_O_5_ hydrolysis, as measured by Bertram and Thornton using mixed NaNO_3_ and NH_4_HSO_4_ particles of 113 nm average radius.^17^ The data points are fit by a resistor model that takes into account the finite size of the aerosol droplets^18,53^ (see Section VIII of the SI). We use *k*_NO_3_^–^_ */k*_H_2_O_ = 17 from Bertram and Thornton^17^ and our range in *k*_NO_3_^–^_ */k*_H_2_O_ to fit the N_2_O_5_ uptake. Figure 8A reveals that the modeled uptake using *k*_NO_3_^–^_ */k*_H_2_O_ between 70 and 230 significantly overestimates nitrate suppression. Compared to the optimized model parameters, our modeled uptake is 2–4 times smaller at 1 M NO_3_^–^ and 3–10 times smaller at 6 M NO_3_^–^. Griffiths et al. also measured N_2_O_5_ uptake onto aerosol and determined a *k*_NO_3_^–^_ */k*_H_2_O_ = 30,^18^ which is larger than the Bertram-Thornton value of 17 but still significantly smaller than our range of 70–230.

**Figure 8:**
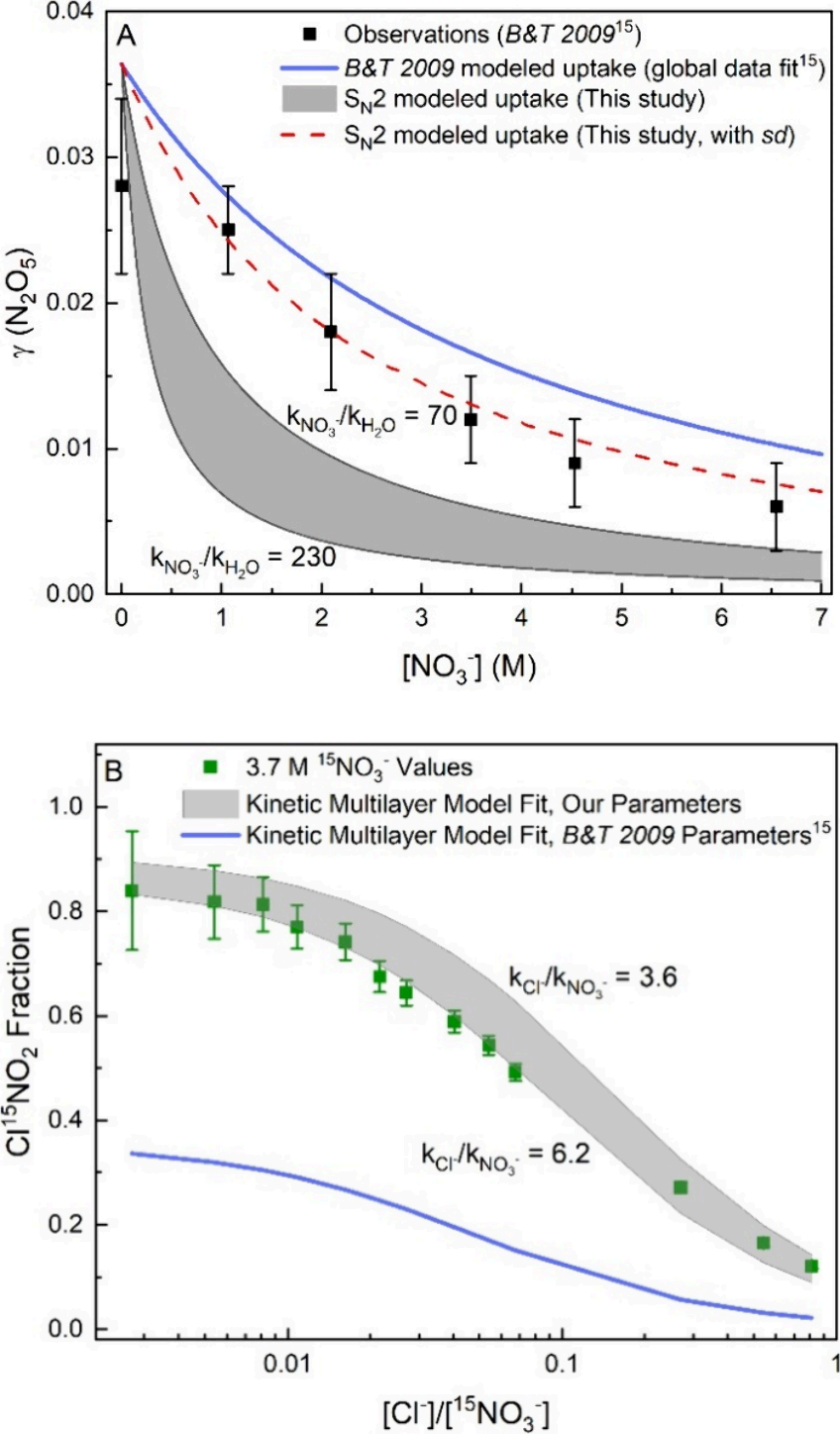
(A) N_2_O_5_ reactive uptake coefficient γ versus NO_3_^–^ concentration. The declining uptake probability reflects the suppression of hydrolysis by added NO_3_^–^. The black squares and error bars are from ref 17 (B&T 2009). The blue curve is fit by the S_N_1 model ([Disp-formula eqR1]–[Disp-formula eqR7]) and *k*_NO_3_^–^_ */k*_H_2_O_ = 17 (ref 17 and SI Section VIII). The gray region, which decreases too sharply, is a fit using the same model and *k*_NO_3_^–^_ */k*_H_2_O_ = 70–230 from our Cl^15^NO_2_ isotope fractions. The red dashed line is a better fit using the S_N_2 model with *k*_NO_3_^–^_ */k*_H_2_O_ = 150 and additional spontaneous N_2_O_5_* deactivation step (*sd*) R-8, such that *k*_sd_ */k*_H_2_O_ = 300 M. See SI section VIII) Cl^15^NO_2_ isotope fractions (from Figure 4B) versus [Cl^–^]/[NO_3_^–^] for 3.7 M Na^15^NO_3_. Our model S_N_2 fits are in gray (SI Section I). The Cl^15^NO_2_ isotope fractions drop with [Cl^–^]/[NO_3_^–^] as chlorination of ^14,14^N_2_O_5_ outcompetes nitration. The blue curve is calculated from the KM model and uses the additional B&T 2009 S_N_1 ratio *k*_Cl^–^_ */k*_NO_3_^–^_ = 29. It severely undercuts the isotope fractions, indicating that the S_N_1 model cannot simultaneously fit the data in both panels.

Conversely, Figure 8B illustrates the discrepancy between the experimental Cl^15^NO_2_ isotope fractions for the 3.7 M Na^15^NO_3_ solution reported in Figure 4B and predictions from the Bertram-Thornton parametrization used above (*k*_Cl^–^_ */k*_NO_3_^–^_ = 29 and *k*_NO_3_^–^_ */k*_H_2_O_ = 17). This parametrization significantly underpredicts the isotope fraction. In the low [Cl^–^]/[^15^NO_3_^–^] ratio range, the fitted isotope fraction is less than half of the experimental fraction for the 3.7 M ^15^NO_3_^–^ solution. The rate constant ratio range of *k*_NO_3_^–^_ */k*_H_2_O_ = 70–230 determined in this study do not align with results from previous studies, while the previously reported rate constant ratio, *k*_NO_3_^–^_ */k*_H_2_O_ = 17, is similarly unable to match our experimental findings.

Figure 8A,B highlight discrepancies between rate parameters that model N_2_O_5_ uptake and those that model Cl^15^NO_2_ isotope fractions. These discrepancies lead us to consider key differences between the previously reported S_N_1 NO_2_^+^ model most often used to parametrize uptake^17,18,23^ and the current S_N_2 model parametrizing isotopic exchange between NO_3_^–^ and N_2_O_5_*. We are able to fit both data sets in Figure 8A,B using the S_N_2 reaction scheme [Disp-formula eqR8]–[Disp-formula eqR11]. This scheme involves both N_2_O_5_* exchange and deactivation by NO_3_^–^ ([Disp-formula eqR11]) and solvent-induced deactivation ([Disp-formula eqR-8]). This latter step, N_2_O_5_* + NO_3_^–^ → N_2_O_5_ + NO_3_^–^, closely parallels the S_N_1 step NO_2_^+^ + NO_3_^–^ → N_2_O_5_ ([Disp-formula eqR-2]). In each mechanism, a ^14,14^N_2_O_5_ molecule immersed in a pool of ^15^NO_3_^–^ will undergo both ^15^NO_3_^–^ exchange and deactivation of the reactive species N_2_O_5_* or NO_2_^+^. The S_N_2 model, however, includes an additional spontaneous deactivation step, N_2_O_5_* → N_2_O_5_ ([Disp-formula eqR-8]), which is likely induced by fluctuations in the position of N_2_O_5_* and/or suppression of charge separation in NO_2_^δ+^NO_3_^δ−^. This spontaneous deactivation is an essential step in the S_N_2 mechanism, as it is the reverse of N_2_O_5_ activation ([Disp-formula eqR8]), but it is necessarily absent in the S_N_1 mechanism, where NO_2_^+^ deactivation occurs only by recombination with NO_3_^–^ ([Disp-formula eqR-2]). While the spontaneous deactivation step cancels out in the calculation of the Cl^15^NO_2_ isotope fraction (as shown in Section IV of the SI), it provides an additional parameter, the rate constant *k*_sd_, in the S_N_2 model that enables fitting of Figure 8A.

The simultaneous fitting in Figure 8A,B is carried out in the SI, Section VIII and Figure S6, using steady-state solutions of the activation and deactivation steps. We find that the uptake data in Figure 8A can be fit using *k*_NO_3_^–^_ */k*_H_2_O_ = 150, as determined above, and by choosing *k*_sd_/*k*_H_2_O_ = 300 M. This additional parameter corresponds to *k*_sd_/(*k*_H_2_O_[H_2_O]) = 5–6 for the ratio of spontaneous N_2_O_5_* deactivation to hydrolysis. For a 1 M NO_3_^–^ and 1 M Cl^–^ solution, the S_N_2 model predicts that N_2_O_5_* is deactivated roughly five times faster than it hydrolyzes, twice as fast as it is nitrated, and one-half as fast as it is chlorinated. The S_N_2 N_2_O_5_* mechanism is thus a plausible replacement for the S_N_1 NO_2_^+^ mechanism, where N_2_O_5_* may be an energetically activated and/or interfacially located species. Our chief caution in promoting this replacement is that the S_N_2 mechanism requires the additional fitting parameter *k*_sd_ for spontaneous deactivation enforced by solvent fluctuations. It will be intriguing to learn if future theory efforts indeed identify N_2_O_5_ deactivation as a key step in controlling the reactivity of N_2_O_5_.

## Conclusions

We have measured the production of isotopically labeled ^14,15^N_2_O_5_,^15,15^N_2_O_5_, and Cl^15^NO_2_ upon flowing ^14,14^N_2_O_5_ over solutions containing Na^15^NO_3_ and NaCl, confirming the existence of chemical exchange between N_2_O_5_ and NO_3_^–^. Thus, nitration of N_2_O_5_ is a normally unobserved reaction that may often occur before N_2_O_5_ evaporates or reacts irreversibly. Using the relative production of Cl^15^NO_2_ and Cl^14^NO_2_, we determined the rate constant ratio between N_2_O_5_ + ^15^NO_3_^–^ and N_2_O_5_ + Cl^–^ to be *k*_Cl^–^_ */k*_NO_3_^–^_ = 2–6 for solutions of 3.7–0.47 M Na^15^NO_3_ over a 0 to 3.0 M [Cl^–^] range. In combination with prior measurements of competitive nitration and hydrolysis of *k*_Cl^–^_/*k*_H_2_O_, we find that *k*_NO_3_^–^_ */k*_H_2_O_ lies between 70 and 230. This range, obtained from isotope ratios of evaporating Cl^14^NO_2_ and Cl^15^NO_2_, does not fit the suppressed uptake of N_2_O_5_ into NaNO_3_ solutions as well as the ratio of *k*_NO_3_^–^_ */k*_H_2_O_ = 17 obtained previously by Bertram and Thornton^17^ when modeling the N_2_O_5_ uptake data directly using the NO_2_^+^ S_N_1 mechanism [Disp-formula eqR1]–[Disp-formula eqR3]. In turn, this S_N_1 mechanism does not fit the Cl^15^NO_2_ isotope fraction measurements. We find that an alternate S_N_2 mechanism involving both spontaneous N_2_O_5_ activation to an energetic or interfacially located N_2_O_5_*, and its deactivation back to N_2_O_5_, can fit both isotope and uptake measurements in nitrate solutions. This model demands an additional rate constant for N_2_O_5_* deactivation that is not present in the S_N_1 NO_2_^+^ mechanism.

We emphasize that, from an empirical perspective, the reduced parameter sets fit to the NO_2_^+^ mechanism in previous studies^1,17,18,23,54^ provide superior descriptions of the kinetics of N_2_O_5_ uptake into Cl^–^/NO_3_^–^/H_2_O solutions over wide concentration ranges. We hope that the isotope experiments reported here, in combination with earlier uptake measurements such as shown in Figure 8A,^17^ will fuel the continuing theoretical development of a time and depth-resolved description of N_2_O_5_ and its reactions with solvent H_2_O and solute ions and surfactants.^30,31,33,37^ This molecular picture will greatly enhance our understanding of gas–liquid reactions generally in which a hydrophobic molecule such as N_2_O_5_ encounters ion and neutral reaction partners across a near-interfacial region potentially spanning tens of Angstroms.^37^

## Supplementary Material



## Notes

Multilayer analysis, rate constants and parameters, and boundary layer characterization used in the kinetic multilayer model (Section I); statistical analysis of N_2_O_5_ signals (Section II); reaction scheme to determine *k*_Cl^–^_ */k*_NO_3_^–^_ (Section III); S_N_2 and S_N_1 steady-state models to determine the ClNO_2_ isotope fraction (Section IV); isotope fractions in the S_N_2 model (Section V); N_2_O_5_ activation and deactivation in the S_N_2 model (Section VI); comparison of activated species in S_N_1 and S_N_2 models (Section VII); uptake calculations for Figure 8 (Section VIII); parametrizing NO_3_^–^, Cl^–^, and H_2_O concentrations (Section IX); and recasting rate constants in terms of activities (Section X) (PDF)
