# Supplementary material for: Heterogeneous Reactions of N2O5 with Nitrate- and Chloride-Containing Solutions: Isotopic Evidence for the Nitration of N2O5
Source: J Phys Chem A. 2025 Nov 10;129(46):10771–84. doi: 10.1021/acs.jpca.5c06048 (PMC12641490; doi:10.1021/acs.jpca.5c06048)
Supplement: Supplementary file 1 [file jp5c06048_si_001.pdf]

Supporting Information for

Heterogeneous Reactions of N_2O_5 with Nitrate and Chloride Containing Solutions: Isotopic Evidence for the Nitration of N_2O_5

Thomas F. Derrah[1], Pascale S. J. Lakey[2], Steven J. Kregel[1], Manabu Shiraiwa[2*], Gilbert M. Nathanson[1*], Timothy H. Bertram[1*]

[1]Department of Chemistry, University of Wisconsin – Madison, Madison, Wisconsin, 53706, United States

[2]Department of Chemistry, University of California – Irvine, Irvine, California 92697, United States

Topic **Page Number**

I. Kinetic Modeling Methods S2

Tables S1 and S2: Reactions, Rate Constants, and Parameters S6-8

II. Statistical Analysis of the N_2O_5 Isotope Signals S11

III. Reaction Scheme to Compute the $k_{Cl^-}/k_{NO_3^-}$ Rate Constant Ratio S13

IV. Three Steady-State S_N2 and S_N1 Liquid-Phase Models for the $ClNO_2$ Isotope Fraction S15

V. N_2O_5 Isotope Exchange Fractions within the S_N2 Deactivation Model S21

VI. Different Activation and Deactivation Steps in N_2O_5 Chlorination and Nitration S22

VII. Equivalence of N_2O_5* and NO_2^+ When NO_3^- Exchange Is the Only Deactivation Step S23

VIII. Size-Dependent Resistor Models for N_2O_5 Reactive Uptake in Relation to Figure 8 S24
(including spontaneous deactivation and mixed activation/deactivation fits to Figure 8A)

IX. Parameterizing NO_3^-, Cl^-, and H_2O Concentrations from E-AIM S29

X. Recasting Rate Constants in Terms of Activities Instead of Molarities S30-31

References S32

I. Kinetic Modeling Methods

Figure S1 shows a schematic diagram of the kinetic multilayer (KM) model used in this work. The model includes flows into and out of the three regions of the flow reactor. The boundary layer above the solution and the bulk solution are treated using a multilayer approach. The model parameters and flux equations are described in detail in two previous publications.[1,2] We assume that the gas phase is well-mixed. Fickian diffusion is assumed to occur in the boundary layer and the solution bulk. Due to the short reacto-diffusive length, the layers in the bulk close to the surface are significantly thinner (160 layers of 4.69 nm thickness near the surface and 40 layers of 34.4 μm thickness deeper in the bulk) so that concentration gradients can be accounted for, and the model has been tested with varying layer numbers to check for convergence. The model includes reversible adsorption to the surface of the solution and reversible partitioning into the solution bulk. Nineteen reactions are included in the solution bulk-phase and at the surface (sorption and quasi-static layers) and are listed in Table S1 below, alongside their rate constants. In the studies performed here, the diffusion and reaction rate constants are set to be the same across all layers, from the outermost sorption layer deep into the bulk, such that only bulk-phase reactions effectively occur throughout the solution (see note 4 in Table S1). The reaction list consists of the reaction of N_2O_5 to form activated isotopically-labeled N_2O_5*, followed by subsequent reactions with NO_3^-, D_2O, or Cl^- to form a variety of isotopically labeled products. The reaction scheme allows the ^{14}N and ^{15}N isotopes to be tracked as different species over time.

Table S1 does not include spontaneous deactivation (R-8 of the main text, such as $^{14,14}N_2O_5^* \rightarrow {}^{14,14}N_2O_5$, k_{sd}) or nitrate-induced deactivation without exchange (such as $^{14,14}N_2O_5^* + {}^{15}NO_3^- \rightarrow {}^{14,14}N_2O_5 + {}^{15}NO_3^-$)) or deactivation of N_2O_5* by Cl^- or Na^+ as separate steps. These missing deactivation reactions do not change the predicted *ratios* of production rates of the various

species (ratios of $ClNO_2$ isotopes) or rate constants ratios because the deactivation steps only alter the concentrations of the N_2O_5* isotopes, which are the common reaction intermediates for hydrolysis and chlorination (see Sections IV, V, VI and VII of this SI). These deactivation steps were omitted in the reaction set in order to reduce the number of fitted parameters to just one rate constant ratio, $k_{Cl^-}/k_{NO_3^-}$. To confirm this claim, we added reaction R-8 to the reaction set at k_{sd}/k_w = 300 M (a fitted value chosen on page S26) and found no change in the predicted $^{15}ClNO_2$ isotope fraction in the KM modeling.

Additional model parameters are listed in Table S2 on page S6 and include Henry's law coefficients, bulk and gas-phase diffusion coefficients, initial surface accommodation coefficients, desorption lifetimes, volumes in the flow reactor, thickness and surface area of the solution, volumetric flow rate, boundary layer length, and the concentration of N_2O_5 in the flow entering the reactor. A set of ordinary differential equations are used to describe the mass balance of each species in the gas phase, in the boundary layer, and in the surface and each bulk layers. These equations are solved numerically using Matlab software.

An important distinction to note is the use of D_2O and H_2O solvents in different experiments. Our $Cl^{15}NO_2$ isotope (Figure 2-6 and 8B and S4-S5) and unlabeled product yield (Figure 7) experiments were run in solutions of D_2O to avoid overlapping peaks in the mass spectra, as described in the main text. All N_2O_5 uptake experiments used H_2O as the solvent and are shown in Figure 8A and S6 from ref 3. To model our isotope and product yield experiments, we combine parameters from previous studies that have used either H_2O or D_2O as solvents. In Table S1, the k_{act} was chosen to match the uptake coefficient of N_2O_5 onto H_2O, which has been measured to be ~0.03.[3] The rate constant ratios for k_{Cl^-}/k_{D_2O} comes from Kregel et al, which was measured from solutions of NaCl in D_2O.[4] We use this ratio to determine the rate constant ratios

$k_{Cl^-}/k_{NO_3^-}$ and $k_{NO_3^-}/k_{D_2O}$ from our model results. In the main text, we convert $k_{NO_3^-}/k_{D_2O}$ to $k_{NO_3^-}/k_{H_2O}$. In Table S2, many of our parameters were determined from theoretical studies using H_2O as a solvent, including the Henry's law constant for $ClNO_2$, derived from density functional theory)[5] and the Henry's law constant for N_2O_5, the aqueous phase diffusion constant for N_2O_5, and the surface accommodation coefficient from MB-Pol simulations.[6] We assume these parameters determined from studies using H_2O are applicable to our study in D_2O, aside from the rate constants of k_{D_2O} and k_{H_2O}, which we distinguish. Kregel et al found k_{Cl^-}/k_{D_2O} is 1.4 times larger than k_{Cl^-}/k_{H_2O}, due to the kinetic isotope effect.[4]

Figure S2 shows the effect of the boundary layer thickness on the relative production of isotopically labeled species. Panel A shows the fraction of labeled N_2O_5 that is doubly-labeled $^{15,15}N_2O_5$. The fraction of $^{15,15}N_2O_5$ is constant across the range in boundary layer thicknesses tested. Panel B shows that the $Cl^{15}NO_2$ fraction is also constant over the boundary layer thickness. These constant values indicate that the depth of the boundary layer does not impact the relative reaction rates.

Figure S3 shows the model results for the concentrations of $^{14,14}N_2O_5$, total $ClNO_2$, $^{14,15}N_2O_5$, and $^{15,15}N_2O_5$ as the height of the boundary layer was varied. Previous experiments predicted a N_2O_5 loss of 20-40% to solution. Using this loss and the model results in Figure S3, we predict the boundary layer thickness in the dual channel flow reactor to be about 0.1 cm, potentially ranging from 0.06 – 0.3 cm. At this 0.1 cm thickness, the model predicts a low concentration of $^{14,15}N_2O_5$ and $^{15,15}N_2O_5$ in the gas phase, in the tens of parts per trillion concentrations. Furthermore, the model predicts a significantly lower concentration of $^{14,15}N_2O_5$ and $^{15,15}N_2O_5$ relative to the amount of N_2O_5 lost or total $ClNO_2$ produced by a factor of 100. As shown in Figure S3B, the modeled ratio of $^{14,15}N_2O_5$ and $^{15,15}N_2O_5$ is strongly dependent on the

assumed boundary layer thickness. Figure 3B also shows that the concentration of $ClNO_2$ is significantly larger than the sum of $^{14,15}N_2O_5$ and $^{15,15}N_2O_5$, due to subsequent reaction of the isotopically labeled gaseous N_2O_5 species after absorption back into solution. This absorption becomes more pronounced as the boundary layer expands. While our measurements agree with the large $ClNO_2$ concentrations predicted by the model and the small concentrations of isotopically labeled N_2O_5 species, we do not explore further model-measurement agreement in the ratio of $^{14,15}N_2O_5$ and $^{15,15}N_2O_5$ due to the very low concentrations observed and predicted in the model analysis.

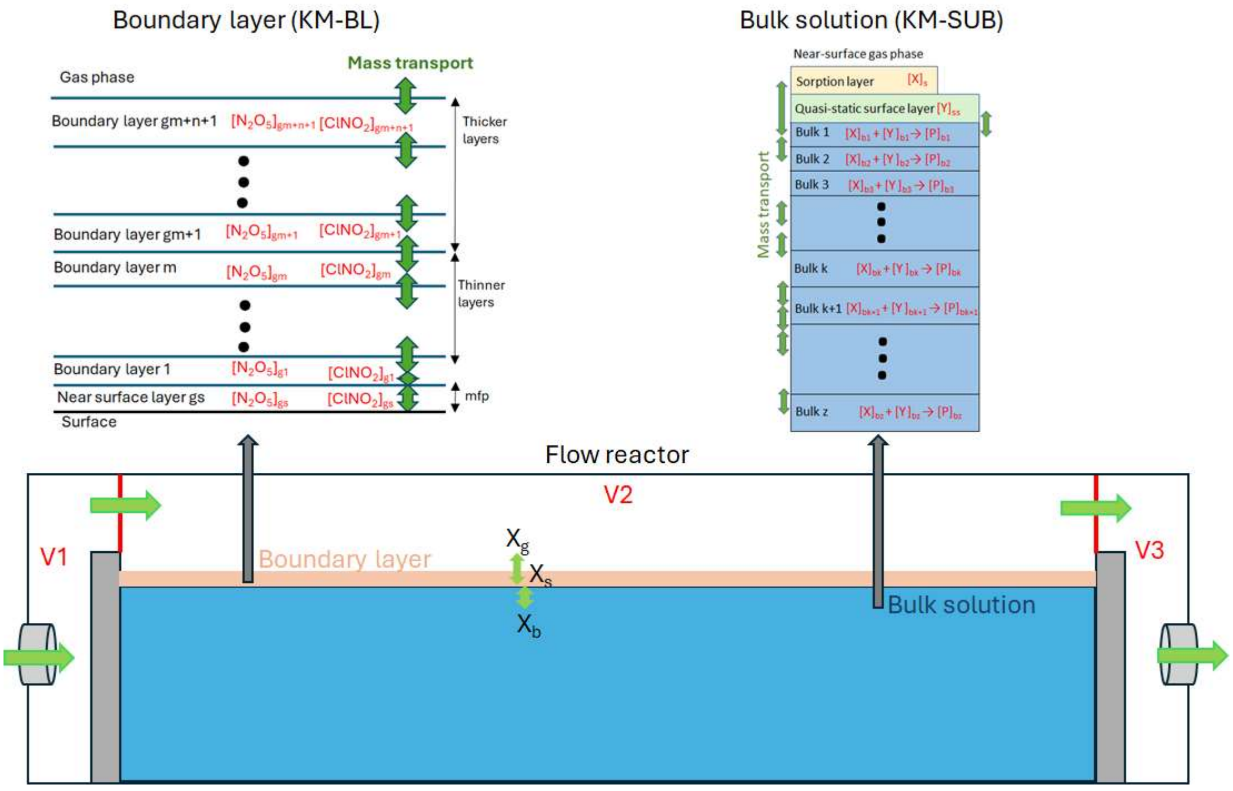

Figure S1: Schematic diagram of the kinetic model used in this work. The model consists of flows through a flow reactor, gas-phase diffusion which is treated using the kinetic multi-layer model of the boundary layer (KM-BL) and surface and bulk mass transport and reactions which are treated using the kinetic multi-layer model of aerosol surface and bulk chemistry (KM-SUB).[1, 2]

Table S1: Reactions and Rate Constants Used in the Kinetic Multi-Layer Model

Reaction number	Reaction (see notes on next page)	Bulk reaction rate constant k values	Reference or additional information
1	$^{14,14}N_2O_{5(aq)} \rightarrow {}^{14,14}N_2O_5{*}_{(aq)}$	4.0×10^5 s^{-1}	$\boldsymbol{k_{act}}$ Required to obtain an uptake coefficient of ~0.03. This value is within the error bars of uptake measurements onto H_2O aerosol from ref 3.
2	$^{14,15}N_2O_{5(aq)} \rightarrow {}^{14,15}N_2O_5{*}_{(aq)}$	4.0×10^5 s^{-1}	
3	$^{15,15}N_2O_{5(aq)} \rightarrow {}^{15,15}N_2O_5{*}_{(aq)}$	4.0×10^5 s^{-1}	
4	$^{14,14}N_2O_5{*}_{(aq)} + {}^{15}NO_3^-{}_{(aq)} \rightarrow {}^{14,15}N_2O_{5(aq)} + {}^{14}NO_3^-{}_{(aq)}$	$(1.3 - 2.2) \times 10^8$ M^{-1} s^{-1}	$\boldsymbol{k_{exd}}$ **or** $\boldsymbol{k_{NO_3^-}}$ Unknown, determined by fitting to the measured $^{15}ClNO_2$ isotope fractions in D_2O. See notes below.
5	$^{14,15}N_2O_5{*}_{(aq)} + {}^{15}NO_3^-{}_{(aq)} \rightarrow {}^{15,15}N_2O_{5(aq)} + {}^{14}NO_3^-{}_{(aq)}$	$(1.3 - 2.2)/2 \times 10^8$ M^{-1} s^{-1}	
6	$^{14,15}N_2O_5{*}_{(aq)} + {}^{15}NO_3^-{}_{(aq)} \rightarrow {}^{14,15}N_2O_{5(aq)} + {}^{15}NO_3^-{}_{(aq)}$	$(1.3 - 2.2)/2 \times 10^8$ M^{-1} s^{-1}	
7	$^{15,15}N_2O_5{*}_{(aq)} + {}^{15}NO_3^-{}_{(aq)} \rightarrow {}^{15,15}N_2O_{5(aq)} + 15NO_3^-{}_{(aq)}$	$(1.3 - 2.2) \times 10^8$ M^{-1} s^{-1}	
8	$^{14,14}N_2O_5{*}_{(aq)} + D_2O_{(l)} \rightarrow 2\ D^+_{(aq)} + 2\ {}^{14}NO_3^-{}_{(aq)}$	4.0×10^7 s^{-1}	$\boldsymbol{k_{D_2O}[D_2O]}$ Chosen to be 100 times larger than k(R1-R3) to ensure that R1-R3 are the rate-limiting steps in the model.
9	$^{14,15}N_2O_5{*}_{(aq)} + D_2O_{(l)} \rightarrow 2\ D^+_{(aq)} + {}^{14}NO_3^-{}_{(aq)} + {}^{15}NO_3^-{}_{(aq)}$	4.0×10^7 s^{-1}	
10	$^{15,15}N_2O_5{*}_{(aq)} + D_2O_{(l)} \rightarrow 2\ D^+_{(aq)} + 2\ {}^{15}NO_3^-{}_{(aq)}$	4.0×10^7 s^{-1}	
11	$^{14,14}N_2O_5{*}_{(aq)} + Cl^-_{(aq)} \rightarrow Cl^{14}NO_{2(aq)} + {}^{14}NO_3^-{}_{(aq)}$	8.0×10^8 M^{-1} s^{-1} or 3.5×10^8 M^{-1} s^{-1}	$\boldsymbol{k_{Cl^-}}$ determined from k_{Cl^-}/k_{D_2O}. Ratio is from Kregel et al (2023)[4] from measurements in D_2O.<br><br>The two values result in k_{Cl^-}/k_W = 1150 and 500, respectively.
12	$^{14,15}N_2O_5{*}_{(aq)} + Cl^-_{(aq)} \rightarrow Cl^{15}NO_{2(aq)} + {}^{14}NO_3^-{}_{(aq)}$	4.0×10^8 M^{-1} s^{-1} or 1.75×10^8 M^{-1} s^{-1}	
13	$^{14,15}N_2O_5{*}_{(aq)} + Cl^-_{(aq)} \rightarrow Cl^{14}NO_{2(aq)} + {}^{15}NO_3^-{}_{(aq)}$	4.0×10^8 M^{-1} s^{-1} or 1.75×10^8 M^{-1} s^{-1}	
14	$^{15,15}N_2O_5{*}_{(aq)} + Cl^-_{(aq)} \rightarrow Cl^{15}NO_{2(aq)} + {}^{15}NO_3^-{}_{(aq)}$	8.0×10^8 M^{-1} s^{-1} or 3.5×10^8 M^{-1} s^{-1}	
15	$^{14,14}N_2O_5{*}_{(aq)} + {}^{14}NO_3^-{}_{(aq)} \rightarrow {}^{14,14}N_2O_{5(aq)} + {}^{14}NO_3^-{}_{(aq)}$	$(1.3 - 2.2) \times 10^8$ M^{-1} s^{-1}	$\boldsymbol{k_{NO_3^-}}$

16	$^{14,15}N_2O_5*_{(aq)} + {}^{14}NO_3^-{}_{(aq)} \rightarrow {}^{14,15}N_2O_{5(aq)} + {}^{14}NO_3^-{}_{(aq)}$	(1.3 – 2.2)/2 × 10^8 M^{-1} s^{-1}	Assumed to have the same value as for reactions 4 – 7.
17	$^{14,15}N_2O_5*_{(aq)} + {}^{14}NO_3^-{}_{(aq)} \rightarrow {}^{14,14}N_2O_{5(aq)} + 15NO_3^-{}_{(aq)}$	(1.3 – 2.2)/ 2 × 10^8 M^{-1} s^{-1}	
18	$^{15,15}N_2O_5*_{(aq)} + {}^{14}NO_3^-{}_{(aq)} \rightarrow {}^{14,15}N_2O_{5(aq)} + {}^{15}NO_3^-{}_{(aq)}$	(1.3 – 2.2) × 10^8 M^{-1} s^{-1}	

Table Notes

1) Rate constants for reactions of $^{1415}N_2O_5*$ with Cl^- and NO_3^- were divided by two with respect to rate constants for reactions of $^{1414}N_2O_5*$ and $^{1515}N_2O_5*$ because there are two distinct products for this mixed isotope species. For example:

$^{1415}N_2O_5* + Cl^-{}_{(aq)} \rightarrow Cl^{15}NO_{2(aq)} + {}^{14}NO_3^-{}_{(aq)}$ (reaction R12)

and

$^{1415}N_2O_5* + Cl^-{}_{(aq)} \rightarrow Cl^{14}NO_{2(aq)} + {}^{15}NO_3^-{}_{(aq)}$ (reaction R13)

The reactions are assumed to occur with equal probabilities and rate constants and always occur in pairs.

2) The first-order rate constant for hydrolysis, $k_{D_2O}[D_2O]$ applies to the hydrolysis reactions R8-R11. The rate constant is set equal to 4.0 × 10^7 s^{-1}. This becomes a second-order rate constant k_{D_2O} = 7.2 × 10^5 M^{-1} s^{-1} for $[D_2O]$ = 55.3 M at 298 K. Using ratios of k_{Cl^-}/k_w = 1150 and 500 yields k_{Cl^-} = 8.3 × 10^7 M^{-1} s^{-1} and 3.6 × 10^7 M^{-1} s^{-1}, respectively. These were rounded to 8.0 × 10^7 M^{-1} s^{-1} and 3.5 × 10^7 M^{-1} s^{-1} for the Kinetic Multi-Layer calculations.

3) Numerical values of the rate constants are required for the simulations, but only their ratios are meaningful. This is because $k_{D_2O}[D_2O]$ for R 8-11 is set arbitrarily to be 100 times k_{act} (R1-3) in order to ensure that k_{act} is the rate-limiting step.

4) The Kinetic Multi-Layer Model naturally incorporates interfacial reactions in the sorption and quasi-static layers. The surface rate coefficients for adsorbed molecules are in units of cm^2 s^{-1} or s^{-1}. However, reactions at the surface were assumed to occur at the same rate as in the bulk and were estimated by dividing the bulk value in cm^3 s^{-1} by the diameter of 1 molecule of one N_2O_5*. This equality ensures that all reactions take place effectively at the bulk-phase rates. We confirmed this result by removing reactions in the sorption and quasi-static layers and found negligible changes in the predicted $^{15}ClNO_2$ isotope fractions. For the parameters chosen here, the large reacto-diffusive length of (D/k_{act}) = 700 Å implies that little reaction occurs in the outermost 10 Å surface region.

Table S2: Parameters Used in the Kinetic Multi-Layer Model

Parameter	Description	Value	Reference or explanation
H_{N2O5}	Henry's law coefficient of N_2O_5	3.0 M atm^{-1}	Cruzeiro et al. (2022)[6] MB-Pol simulations using H_2O
H_{ClNO2}	Henry's law coefficient of $ClNO_2$ (0.04 M atm^{-1} theory estimate[13])	0.024 M atm^{-1}	Behnke et al. (1997)[5]
$D_{g,N2O5}$	Gas-phase diffusion coefficient of N_2O_5	0.11 cm^2 s^{-1}	Tang et al. (2014)[7]
$D_{g,ClNO2}$	Gas-phase diffusion coefficient of $ClNO_2$	0.12 cm^2 s^{-1}	Tang et al. (2014)[7]
$D_{b,N2O5}$	Bulk diffusion coefficient of N_2O_5	1.9×10^{-5} cm^2 s^{-1}	Cruzeiro et al. (2022)[6] MB-Pol simulations using H_2O
$D_{b,ClNO2}$	Bulk diffusion coefficient of $ClNO_2$	1.9×10^{-5} cm^2 s^{-1}	Set equal to N_2O_5 value
$D_{b,NO3-}$	Bulk diffusion coefficient of NO_3^-	1.8×10^{-5} cm^2 s^{-1}	Estimated using the EPA online diffusion coefficient calculator (Note that small changes in these values do not impact results)
$D_{b,Cl-}$	Bulk diffusion coefficient of Cl^-	2.3×10^{-5} cm^2 s^{-1}	
$D_{b,\ N2O5*}$	Bulk diffusion coefficient of N_2O_5*	1×10^{-5} cm^2 s^{-1}	
$\alpha_{s,0,all}$ (also called S)	Initial surface accommodation coefficient of N_2O_5 and $ClNO_2$	0.96	Cruzeiro et al. (2022)[6] MB-Pol simulations using H_2O. Assumed to be the same for $ClNO_2$
$\tau_{d,all}$	Desorption lifetime of N_2O_5 and $ClNO_2$	1 ns	Estimated value/not sensitive to < 10 ns
V_1 and V_3	Volume 1 and Volume 3 as depicted in Figure S1	0.97 cm^3	Experimental value
V_2	Volume 2 as depicted in Figure S1	11.34 cm^3	Experimental value (includes the boundary layer)
δ_{sol}	Total thickness of the solution	1.38 cm	Experimental value
A_{sol}	Total surface area of the solution	14.5 cm^2	Experimental value
φ	Volumetric flow rate	25 cm^3 s^{-1}	Experimental value
δ_{BL}	Total thickness of the boundary layer	0.1 cm	Determined by the approximate decrease in N_2O_5 exiting the reactor.
$[N_2O_5]_{g,in}$	Concentration of N_2O_5 in the flow entering the reactor	20 ppb	Experimental value

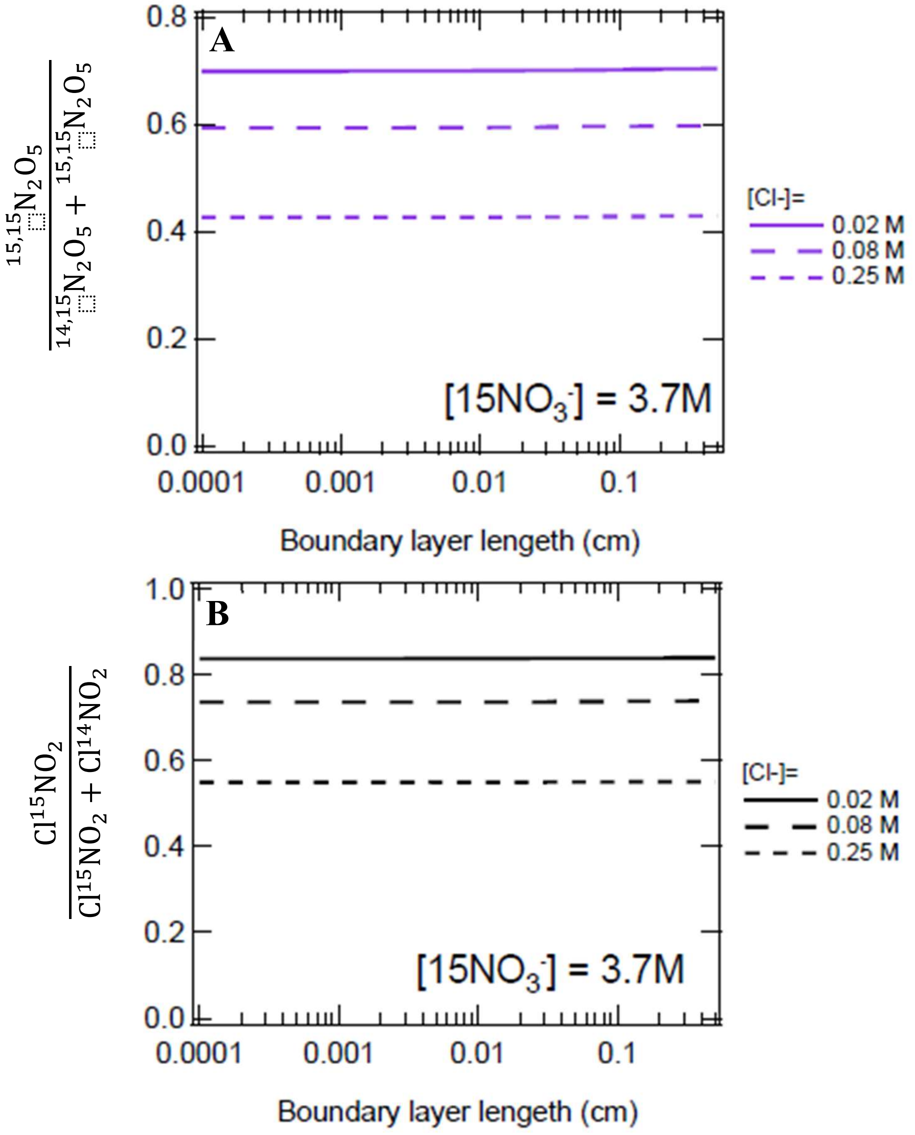

Figure S2. Plots of the fraction of labeled N_2O_5 that is $^{15,15}N_2O_5$ (A) and the heavy $Cl^{15}NO_2$ fraction (B) as the boundary layer length is varied. Model results were collected at 0.02, 0.08, and 0.25 M Cl^- with 3.7 M $^{15}NO_3^-$.

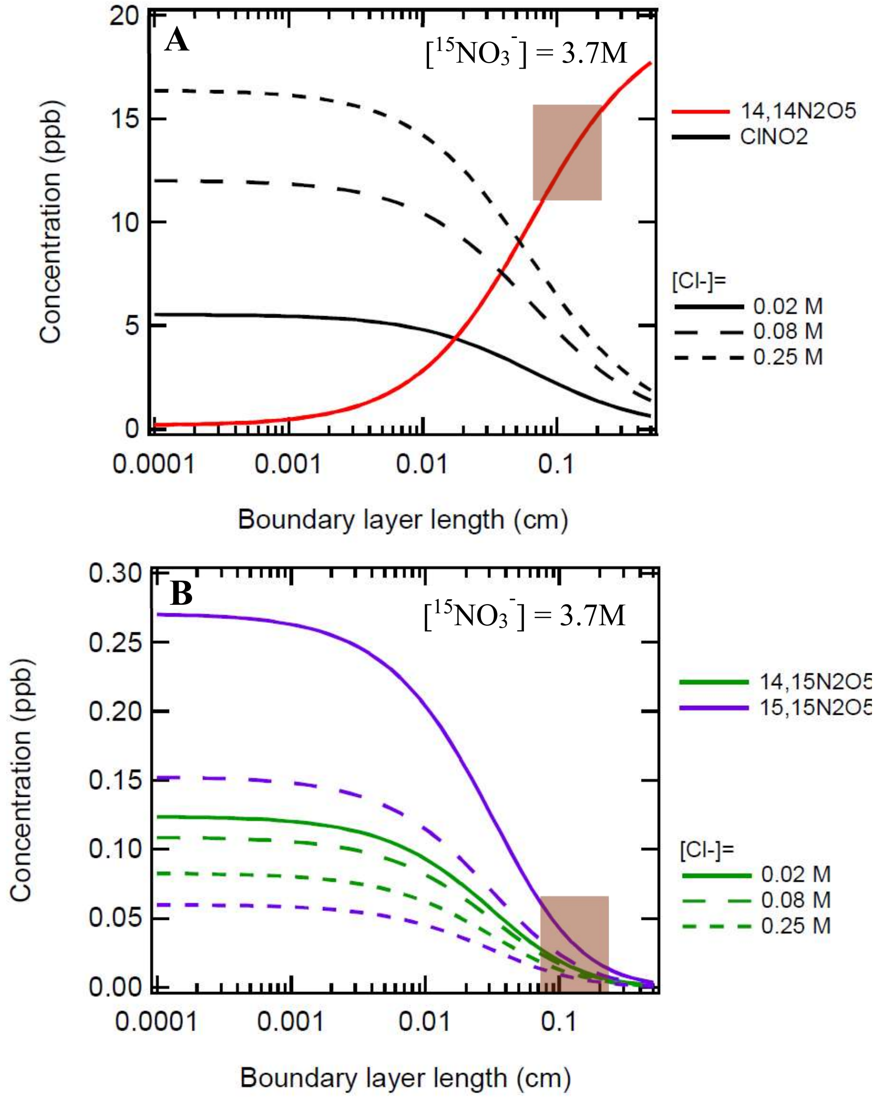

Figure S3. Plots of the concentrations (in ppb) of $^{14,14}N_2O_5$ and total $ClNO_2$ (A), and $^{14,15}N_2O_5$ and $^{15,15}N_2O_5$ (B) species determined from the model as the boundary layer length is varied. The concentrations of gaseous species are given at 3 different Cl^- concentrations. The initial $^{14,14}N_2O_5$ concentration was 20 ppb. The transparent orange box indicates the expected boundary layer regime for experiments in which there is a 20-40% decrease in N_2O_5.

II. Statistical Analysis of the N_2O_5 Isotope Signals

This section describes the statistical analysis of the N_2O_5 isotope signals in Figure 3 of the main text. We first performed a two-sample Student's *t* test on the $^{14,15}N_2O_5$ time series in Figure 3A. We calculate a *t* statistic of 28.3, which is larger than the *t* value for significance for 240 degrees of freedom at 95% confidence (t = 1.97), verifying that the signals of $I(^{14,15}N_2O_5)^-$ from the different solutions are statistically different. The signal for $I(^{15,15}N_2O_5)^-$ from the same solution has a smaller visual difference (Figure 3B). Performing the same statistics test results in a *t* statistic of 10.5, which also indicates this signal is statistically different between the two solutions. Figures 3D and 3E show the $I(^{14,15}N_2O_5)^-$ and $I(^{15,15}N_2O_5)^-$ signals from flow over the solution of 0.47 M $Na^{15}NO_3$ / 0.1 M NaCl and the reference solution, with a smaller magnitude in the signals as well as a smaller difference between the solutions. However, performing the statistics test on $I(^{15,15}N_2O_5)^-$ on Figure 3E results in a *t* statistic of 5.6, which is still greater than the *t* value for 95% confidence (t = 1.97), indicating there is a statistically significant difference in the signal of $I(^{15,15}N_2O_5)^-$ between the two solutions.

Figure S4 shows histograms of the $^{14,15}N_2O_5$ and $^{15,15}N_2O_5$ signals in Figure 3A, 3B, 3D, and 3F of the main text. These histograms include 120 data points of $^{14,15}N_2O_5$ and $^{15,15}N_2O_5$ over a saturated NaCl in D_2O solution and over a mixed $Na^{15}NO_3$ and NaCl in D_2O solution. The histograms are separated into bins of size 20, with solid lines corresponding to the normal distribution of the measured signals. The shaded regions are 1 standard deviation above and below the mean. In both plots of $^{15,15}N_2O_5$, the one standard deviation regions overlap for both the saturated NaCl solution and mixed $Na^{15}NO_3$ / NaCl solution. They also overlap for $^{14,15}N_2O_5$ signals from the saturated NaCl and the 0.47 M $Na^{15}NO_3$ / 0.1 M NaCl solutions, but we saw no overlap when comparing the signals from the saturated NaCl and 3.7 M $Na^{15}NO_3$ / 0.02 M NaCl

solutions. While all four plots reveal statistically significant differences between the reference and sample solutions at the 95% confidence level, the evidence for production of $^{14,15}N_2O_5$ from the 3.7 M $Na^{15}NO_3$ solution is particularly robust.

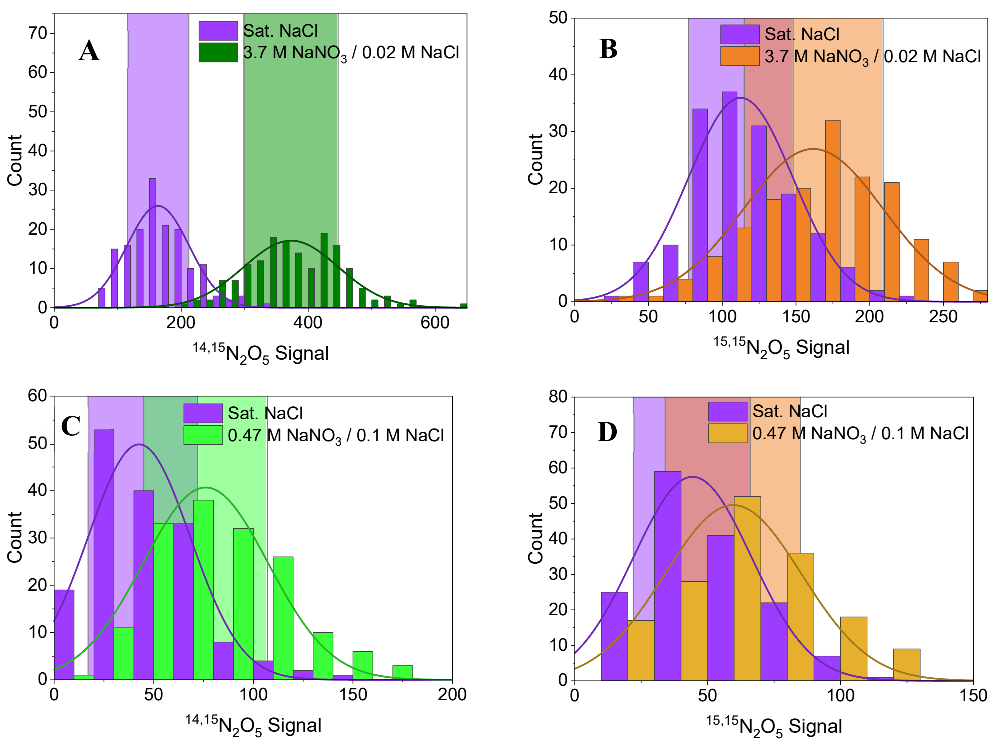

Figure S4. Histograms of $^{14,15}N_2O_5$ (A and C) and $^{15,15}N_2O_5$ (B and D) signals from N_2O_5 flow over saturated NaCl in D_2O solution and over mixed $Na^{15}NO_3$ and NaCl in D_2O solution. The bin size of the histogram is 20. The solid line is the normal distribution of the data from each solution. The shaded regions correspond to one standard deviation around the mean.

III. Reaction Scheme to Compute the $k_{\mathrm{Cl}^-}/k_{\mathrm{NO}_3^-}$ Rate Constant Ratio

The Introduction in the main text identifies two distinct mechanisms that enable N_2O_5 reactive uptake to be consistent with the observed lack of dependence on the NaCl concentration. These schemes are the S_N1 mechanism[3,8,9] invoking NO_2^+ and the S_N2 mechanism invoking a charge-fluctuating[10,11] or interfacially located N_2O_5* as the activated reactive species.[12,13] Kinetically, the difference between these two species is that NO_2^+ can only be deactivated by recombination with NO_3^- to produce N_2O_5, while N_2O_5* can be deactivated by interactions with solvent water molecules and solute species NO_3^-, Cl^-, and Na^+. Importantly, the rate constant ratio $k_{\mathrm{Cl}^-}/k_{\mathrm{NO}_3^-}$ we seek to determine does not depend on the values of these deactivation rate constants or even their presence as long as N_2O_5* is the same species reacting with H_2O, Cl^-, and NO_3^- (see sections IV, V, and VI of this SI for a detailed discussion). In the limiting case in which N_2O_5* deactivation only occurs through chemical exchange between NO_3^- and N_2O_5*, the two models become identical and NO_2^+ and N_2O_5* become interconvertible, as discussed in section VI. Because we can only extract rate constant ratios from the isotope fractions, and because we do not know the values of the deactivation rates (R-8 and R11 in the main text) or the precise nature of N_2O_5*, we choose to extract the $k_{\mathrm{Cl}^-}/k_{\mathrm{NO}_3^-}$ ratio using this limited nitrate deactivation mechanism and its smaller parameter set for kinetic modeling. We also chose this approach to match the S_N1 NO_2^+ mechanism used by previous researchers so that we may compare rate constant ratios in the discussion of Figure 8 in the main text.

The kinetic model in Figure S1 requires computations involving absolute rate constants for this limited parameter set. They are listed in Table S1, but the absolute values of k_w, k_{Cl^-}, and $k_{\mathrm{NO}_3^-}$ do not need to be known to determine the ratio $k_{\mathrm{Cl}^-}/k_{\mathrm{NO}_3^-}$. Relative values of these rate constants are chosen in the following way. The N_2O_5 rate-limiting activation rate constant k is

determined from the bulk-phase resistor equation, ($1/\gamma = 1/\alpha + \langle v\rangle/(4RT(Dk)^{1/2})$),[14] for a typical reactive uptake $\gamma = 0.03$ in H_2O solvent,[3,15,16] Henry's law solubility $H = 3.0$ M/atm, diffusion coefficient $D = 1.9\times10^{-5}$ cm^2/s, average speed $\langle v\rangle = (8RT/\pi m)^{1/2}$, and mass accommodation coefficient $\alpha = 0.96$.[6] These parameters are taken from ref [6] and yield $k = 4\times10^5$/s for N_2O_5 activation into N_2O_5*. The absolute value of this rate constant is valid within the bulk-phase diffusion-limited framework for an infinitely deep solution.[14] The bulk-phase N_2O_5 reacto-diffusive length, $(D/k)^{1/2}$, is approximately 70 nm based on these choices. The hydrolysis rate constant k_w is then set to be 100 times greater, 4×10^7/s, to ensure that it is not rate limiting. Lastly, the chlorination rate constant k_{Cl^-} is set to 8×10^8/M/s or 4×10^8/M/s using the k_{Cl^-}/k_w ratio of 1100 (for the 0.0054 to 0.21 M NaCl) or 500 (for 0.54 to 2.4 M NaCl) determined by Kregel et al in D_2O.[4] We only extract the rate constant ratio $k_{Cl^-}/k_{NO_3^-}$ and do not recommend using the absolute values of k_w, k_{Cl^-} or $k_{NO_3^-}$ listed in Table S1 for further calculations. The rate constants *ratios* are robustly measured and listed in Table 1 of the main text.

We note that $k = 4\times10^5$/s is replaced by 11.5×10^5/s in section VII to reproduce the measured uptake of $\gamma = 0.03$ at $[NaNO_3] = 0$ M for 113 nm average radius particles used in the cited study.[3] Its exact value does not impact the analysis of the $Cl^{15}NO_2$ isotope fraction, but it must be well chosen to model N_2O_5 uptake data. A careful study in Gaston and Thornton[17] examines the particle size dependence of N_2O_5 hydrolysis and chlorination and concludes that k is close to 10×10^5/s, similar to the value of 11.5×10^5/s obtained in ref[3] and used in Section VII. Ref[17] also concludes from uptake measurements that k_{Cl^-}/k_{H_2O} lies within 700 to 1000, which is somewhat higher than our estimates of 350 to 800 from product yields after applying the D_2O/H_2O correction factor of 1.4[4] to lower the first row of entries in Table 2 of the main text. This discrepancy is a topic that deserves future study as a function of NaCl concentration.

IV. Three Steady-State Liquid-Phase Models for the $ClNO_2$ Isotope Fraction

The value and meaning of the rate constant ratio $k_{Cl^-}/k_{NO_3^-}$ extracted from the $ClNO_2$ isotope fractions depends on the chosen mechanism. We describe here three mechanisms that create and destroy N_2O_5* or NO_2^+. Remarkably, they each predict the same functional form for the isotope fraction. These mechanisms are presented below.

Steady-state solutions for N_2O_5 conversion into $ClNO_2$ can be derived analytically in the liquid phase. These calculations do not account for N_2O_5 or $ClNO_2$ entry or evaporation, interfacial processes, or boundary layer effects. They are obtained by applying the steady-state approximation to the following species, whose time derivatives are set to zero: $^{14,14}N_2O_5$*, $^{14,15}N_2O_5$*, $^{15,15}N_2O_5$*, $^{14,15}N_2O_5$, $^{15,15}N_2O_5$ in the S_N2 models and $^{14}NO_2^+$, $^{15}NO_2^+$ in the S_N1 model. These species are the reaction intermediates that couple the initial $^{14,14}N_2O_5$ and $^{15}NO_3^-$ reactants and final $Cl^{14}NO_2$ and $Cl^{15}NO_2$ products.

A) Water and Ion-Induced Deactivation of N_2O_5*

This S_N2 mechanism, presented in the main text, allows $^{14,14}N_2O_5$* to be deactivated by solvent water molecules and $^{15}NO_3^-$ ions (with analogous steps for $^{14,15}N_2O_5$* and $^{15,15}N_2O_5$*). It also permits activation <u>and</u> deactivation by dissolved ions $X^{-/+}$:

$^{14,14}N_2O_5 \rightarrow {}^{14,14}N_2O_5^*$	spontaneous (water-induced) **act**ivation $k_{\mathbf{act}}$ (same for 14,15 and 15,15 isotopes)
$^{14,14}N_2O_5 + X^{-/+} \rightarrow {}^{14,14}N_2O_5^* + X^{-/+}$	$X^{-/+} = Cl^-/^{15}NO_3^-/Na^+$ for **ion act**ivation k_{ionact} (same for 14,15 and 15,15 isotopes)
$^{14,14}N_2O_5^* \rightarrow {}^{14,14}N_2O_5$	**s**pontaneous (water-induced) **d**eactivation $k_{\mathbf{sd}}$ (same for 14,15 and 15,15 isotopes)
$^{14,14}N_2O_5^* + {}^{15}NO_3^- \rightarrow {}^{14,14}N_2O_5 + {}^{15}NO_3^-$	**n**on-**r**eactive $^{15}NO_3^-$ **d**eactivation $k_{\mathbf{nrd}}$ (same for 14,15 and 15,15 isotopes)
$^{14,14}N_2O_5^* + X^{-/+} \rightarrow {}^{14,14}N_2O_5 + X^{-/+}$	$X^{-/+} = Cl^-/Na^+$ non-reactive ion deactivation use same $k_{\mathbf{nrd}}$ (same for 14,15/15,15 isotopes)

$^{14,14}N_2O_5^* + {}^{15}NO_3^- \rightarrow {}^{14,15}N_2O_5 + {}^{14}NO_3^-$	nitrate **ex**change and **d**eactivation $k_{\mathbf{exd}}$ (also called $k_{NO_3^-}$)
$^{14,14}N_2O_5^* + Cl^- \rightarrow Cl^{14}NO_2 + {}^{14}NO_3^-$	chlorination k_{Cl^-}
$^{14,14}N_2O_5^* + H_2O \rightarrow 2\,H^+ + 2\,{}^{14}NO_3^-$	hydrolysis k_w (w refers to H_2O or D_2O)
$^{14,15}N_2O_5^* + {}^{15}NO_3^- \rightarrow {}^{15,15}N_2O_5 + {}^{14}NO_3^-$	$k_{exd}/2$ (exchange ^{14}N to ^{15}N)
$^{14,15}N_2O_5^* + {}^{15}NO_3^- \rightarrow {}^{15,15}N_2O_5 + {}^{14}NO_3^-$	$k_{exd}/2$ (exchange of ^{15}N to ^{15}N)
$^{15,15}N_2O_5^* + {}^{15}NO_3^- \rightarrow {}^{15,15}N_2O_5 + {}^{14}NO_3^-$	k_{exd} (exchange of ^{15}N to ^{15}N)
$^{14,15}N_2O_5^* + Cl^- \rightarrow Cl^{14}NO_2 + {}^{15}NO_3^-$	chlorination $k_{Cl^-}/2$
$^{14,15}N_2O_5^* + Cl^- \rightarrow Cl^{15}NO_2 + {}^{14}NO_3^-$	chlorination $k_{Cl^-}/2$
$^{14,15}N_2O_5^* + H_2O \rightarrow 2\,H^+ + {}^{14}NO_3^- + {}^{15}NO_3^-$	hydrolysis k_w (w refers to H_2O or D_2O)
$^{15,15}N_2O_5^* + Cl^- \rightarrow Cl^{15}NO_2 + {}^{15}NO_3^-$	chlorination k_{Cl^-}
$^{15,15}N_2O_5^* + H_2O \rightarrow 2\,H^+ + 2\,{}^{15}NO_3^-$	hydrolysis k_w (w refers to H_2O or D_2O)

As one example, the steady-state equation for $^{14,14}N_2O_5^*$ is given by:

$$d[^{14,14}N_2O_5^*]/dt = 0 = \text{activation - deactivation} = \{k_{act} + k_{ionact}[\text{all ions}]\}[^{14,14}N_2O_5] - \{(k_{nrd} + k_{Cl^-})[Cl^-] + k_w[H_2O] + (k_{exd} + k_{nrd})[^{15}NO_3^-] + k_{sd} + k_{nrd}[Na^+]\}[^{14,14}N_2O_5^*]$$

This particular model differs from the model in Table S-1 by including spontaneous deactivation k_{sd} by the solvent and ion-induced non-reactive deactivation k_{nrd}.

The steady-state solution for the $Cl^{15}NO_2$ isotope fraction in D_2O is found to be:

$$[Cl^{15}NO_2]/([Cl^{15}NO_2] + [Cl^{14}NO_2]) = 1/(1 + 2y) \qquad \text{S-1}$$

where

$$y = (k_{Cl^-}/k_{exd})([Cl^-]/[^{15}NO_3^-]) + (k_{D_2O}/k_{exd})([D_2O]/[^{15}NO_3^-]) \qquad \text{S-2}$$

The factor of 2 in front of y in eq S-1 arises because $^{15}NO_3^-$ statistically converts $^{14,15}N_2O_5^*$ into $^{15,15}N_2O_5^*$ in half of the nitration reactions. Equation S-2 reveals that the $Cl^{15}NO_2$ isotope fraction

depends on ratios of concentrations and rate constants but not on their individual values. This equation does not involve the rate constants k_{act}, k_{sd}, k_{nrd}, or k_{ionact} for activation of N_2O_5 or deactivation of N_2O_5*, as they cancel in the fraction (eq S-1). We therefore cannot gauge the nature or magnitude of these rate constants from the isotope ratios, and they are not included in the model in Table S-1. Equation S-2 instead reflects the competition between chlorination and nitration of N_2O_5* in the first term (that depends on $[Cl^-]/[^{15}NO_3^-]$) and between hydrolysis and nitration in the second term (that depends on $[D_2O]/[^{15}NO_3^-]$). This second term is present because hydrolysis reduces the fractions of $^{14,15}N_2O_5$ and $^{15,15}N_2O_5$ that feed $Cl^{15}NO_2$ production.

Figure S5 below shows the fits of equations S-1 and S-2 to the $Cl^{15}NO_2$ isotope fractions in D_2O for three different water/nitrate ratios at 0.47, 1.35, and 3.7 M $^{15}NO_3^-$ when plotted against $[Cl^-]/[^{15}NO_3^-]$. This graph is the same as Figure 4B in the main text but now with added fits.

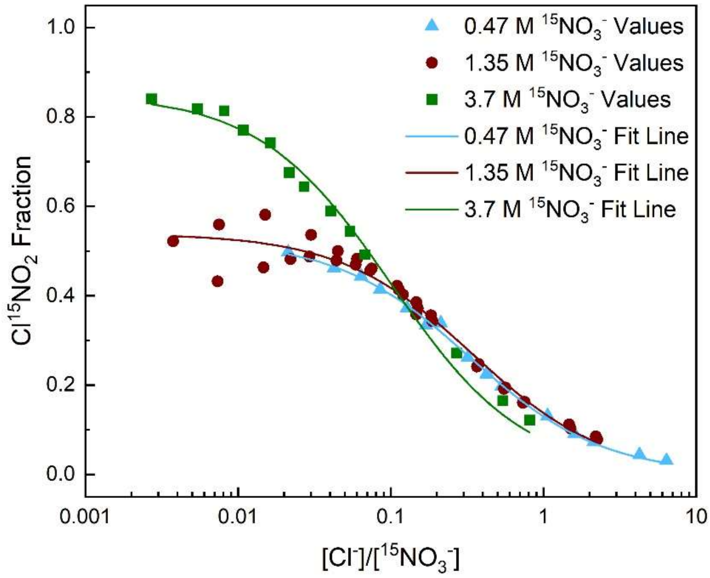

Figure S5. $Cl^{15}NO_2$ isotope fractions for 0.47 M $^{15}NO_3^-$ (blue triangles), 1.35 M $^{15}NO_3^-$ (red circles) and 3.7 M $^{15}NO_3^-$ (green squares) plotted against the $[Cl^-]/[^{15}NO_3^-]$ mole ratio. The fits use eqs S-1 and S-2 for 0.47 M $^{15}NO_3^-$ (blue line), 1.35 M $^{15}NO_3^-$ (red line) and M $^{15}NO_3^-$ (green line).

The data and fits reveal two low and high Cl^- concentration regimes: at low $[Cl^-]$, the second term in y dominates and $[D_2O]/[^{15}NO_3^-]$ controls the isotope fraction. In contrast, at high $[Cl^-]$, the three data sets coalesce into a single curve controlled by $[Cl^-]/[^{15}NO_3^-]$, as expected from the first term in y.

From the fits in Figure S5, we extract $k_{Cl^-}/k_{NO_3^-}$ (or k_{Cl^-}/k_{exd}) ratios of 2.9, 2.7, and 5.8 for 0.47, 1.35, and 3.7 M $^{15}NO_3^-$ respectively. These ratio values fall within the range of 2.2 – 6.2 determined by our kinetic multilayer model in the main text. In addition, we determine $k_{NO_3^-}/k_w$ ratios of 90, 250, and 150 for 0.47, 1.35, and 3.7 M $^{15}NO_3^-$ that also fall within the multilayer model analysis of 100–320. These values are listed in Table S3 below, which incorporates Table I in the main text. The good agreement with the full KMM analysis presented in Section 1 of this SI indicates that the steady-state model captures the essential dependence on the $[Cl^-]/[NO_3^-]$ and $[NO_3^-]/[H_2O]$ ratios. Note that $k_{NO_3^-}$ in Table S3 is the same as k_{exd} in the equations above (page S16 and eq S-2).

Table S3. Rate Constant Ratios from Full and Steady-State Analyses

Rate Constant Ratio	KM: high k_{Cl^-}/k_{D_2O}	KM: low k_{Cl^-}/k_{D_2O}	Steady State S-1
k_{Cl^-}/k_{D_2O}	1150 ± 90	500	---
$k_{Cl^-}/k_{NO_3^-}$	3.6 – 6.2	2.2 – 5.0	2.7, 2.9, 5.8
$k_{NO_3^-}/k_{D_2O}$	190 – 320	100 – 230	90, 150, 250
$k_{NO_3^-}/k_{H_2O}$	130 – 230	70 – 160	64, 110, 180

B) Nitrate Exchange that Maintains Activation

In the opposite extreme, we consider a restricted mechanism in which nitrate exchange maintains the activated state N_2O_5* and does not deactivate it. There are no pathways other than chlorination or hydrolysis that deactivate (destroy) $^{14,15}N_2O_5$* or $^{15,15}N_2O_5$*. Thus, there are no reactions that lead to inactivated $^{14,15}N_2O_5$ or $^{15,15}N_2O_5$, which is possible in the mechanism above. In this case, the key reactions are:

$^{14,14}N_2O_5^* \rightarrow {}^{14,14}N_2O_5^*$ activation k_{act}

$^{14,14}N_2O_5^* + {}^{15}NO_3^- \rightarrow {}^{14,15}N_2O_5^* + {}^{14}NO_3^-$ nitrate **ex**change maintaining **a**ctivation $k_{\mathbf{exa}}$

$^{14,15}N_2O_5^* + {}^{15}NO_3^- \rightarrow {}^{15,15}N_2O_5^* + {}^{14}NO_3^-$ $k_{exa}/2$

$^{14,14}N_2O_5^* + Cl^- \rightarrow Cl^{14}NO_2 + {}^{14}NO_3^-$ chlorination k_{Cl^-}(ditto for $^{14,15}N_2O_5$* and $^{15,15}N_2O_5$*)

$^{14,14}N_2O_5^* + H_2O \rightarrow 2\,H^+ + 2\,{}^{14}NO_3^-$ hydrolysis k_w (ditto for $^{14,15}N_2O_5$* and $^{15,15}N_2O_5$*)

A sample steady-state approximation is

$$d[^{14,14}N_2O_5^*]/dt = 0 = k_{act}[^{14,14}N_2O_5] - \{k_{Cl^-}[Cl^-] + k_w[H_2O] + k_{exa}[^{15}NO_3^-]\}[^{14,14}N_2O_5^*]$$

The combined steady-state approximations lead to a functionally identical solution as above,

$$[Cl^{15}NO_2]/([Cl^{15}NO_2] + [Cl^{14}NO_2]) = 1/(1 + 2y) \qquad \text{S-3}$$

where

$$y = (k_{Cl^-}/\,k_{exa})([Cl^-]/[^{15}NO_3^-]) + (k_{D_2O}/\,k_{exa})([D_2O]/[^{15}NO_3^-]) \qquad \text{S-4}$$

Thus, both mechanisms can fit the data in the same way, and we cannot discriminate between the two mechanisms based on the $^{15}ClNO_2$ isotope fraction alone. In this mechanism, $^{15}NO_3^-$ acts to convert $^{14,14}N_2O_5$ to $^{14,15}N_2O_5$ and $^{15,15}N_2O_5$ and so controls the isotope fraction. However, because NO_3^- cannot deactivate N_2O_5*, there is no nitrate suppression that reduces uptake upon adding NO_3^-. This mechanism alone can therefore not explain the nitrate effect in Figure 8 in the main text.

C) S_N1 NO_2^+ Ionization

This traditional mechanism is described in the Introduction. Its distinguishing feature is that the activated state is NO_2^+ and that it can only be deactivated by recombination with NO_3^-:

$^{14,14}N_2O_5 \rightarrow {}^{14}NO_2^+ + {}^{14}NO_3^-$	ionization k_{ion}
$^{15,15}N_2O_5 \rightarrow {}^{15}NO_2^+ + {}^{15}NO_3^-$	ionization k_{ion}
$^{14,15}N_2O_5 \rightarrow {}^{14}NO_2^+ + {}^{15}NO_3^-$	$k_{ion}/2$
$^{14,15}N_2O_5 \rightarrow {}^{15}NO_2^+ + {}^{14}NO_3^-$	$k_{ion}/2$
$^{14}NO_2^+ + {}^{15}NO_3^- \rightarrow {}^{14,15}N_2O_5$	recombination k_{rec}
$^{15}NO_2^+ + {}^{15}NO_3^- \rightarrow {}^{15,15}N_2O_5$	recombination k_{rec}
$^{14}NO_2^+ + Cl^- \rightarrow Cl^{14}NO_2 + {}^{14}NO_3^-$	chlorination k_{Cl^-} (ditto for $^{15}NO_2^+$)
$^{14}NO_2^+ + H_2O \rightarrow 2\,H^+ + 2\,{}^{14}NO_3^-$	hydrolysis k_w (ditto for $^{15}NO_2^+$)

The steady state solution for the $Cl^{15}NO_2$ isotope fraction is again 1/(1 + 2y), where

$y = (k_{Cl^-}/k_{rec})([Cl^-]/[^{15}NO_3^-]) + (k_{D_2O}/k_{rec})([D_2O]/[^{15}NO_3^-])$

The identical functional form in each mechanism prohibits us from discriminating among them based solely on $Cl^{15}NO_2$ isotope fractions. We emphasize that the rate constants themselves have different meanings in each case. In particular, k_{act} and k_{ion} prepare N_2O_5* and NO_2^+, respectively, while k_{exd}, k_{exa}, and k_{rec} refer to nitrate exchange and deactivation of N_2O_5*, nitrate exchange that maintains activation of N_2O_5*, and recombination of NO_2^+ and NO_3^- those leaves behind a deactivated N_2O_5. Our use of $k_{NO_3^-}$in the main text refers explicitly to a nitrate exchange event, while in nearly all past studies, $k_{NO_3^-}$ refers to the S_N1 $NO_2^+ + NO_3^-$ recombination k_{rec}.

Lastly, a mechanism involving reaction with non-activated N_2O_5 also predicts 1/(1 + 2y), but this assumption removes the essential rate-limiting activation step described in the Introduction.

V. N_2O_5 Isotope Exchange Fractions within the S_N2 Deactivation Model

The S_N2 model can be used to calculate the fractions of each isotopic species and the fraction of $^{14,14}N_2O_5$ molecules that undergo $^{15}NO_3^-$ exchange before chlorination or hydrolysis. The detailed mechanism is listed on pages S13 and S14.

We define the fraction of $^{14,14}N_2O_5$ molecules that undergo exchange before chlorination or hydrolysis as one minus the fraction of $Cl^{14}NO_2$ or $2H^{14}NO_3$ produced from $^{14,14}N_2O_5$ molecules (and not from $^{14,15}N_2O_5$). The steady-state solution for all intermediate species yields:

$y = (k_{Cl^-}/k_{exd})([Cl^-]/[^{15}NO_3^-]) + (k_{H_2O}/k_{exd})([H_2O]/[^{15}NO_3^-])$ (same as eq S-2)

and

f_{exch} (fraction of $^{14,14}N_2O_5$ that exchange at least once before Cl^- or H_2O attack) = $1/(1 + y)$

$Cl^{15}NO_2$ isotope fraction = $1/(1 + 2y)$ (see page S16)

$^{14,14}N_2O_5$ fraction = $y/(1 + y)$

$^{14,15}N_2O_5$ fraction = $2y/\{(1 + 2y)(1 + y)\}$

$^{15,15}N_2O_5$ fraction = $1/\{(1 + 2y)(1 + y)\}$

The f_{exch} and $Cl^{15}NO_2$ fractions are not the same because some $Cl^{14}NO_2$ may come from a single-exchanged $^{14,15}N_2O_5$, which is counted as an exchanged molecule in f_{exch}.

A $Cl^{15}NO_2$ fraction of 1/2 corresponds to $y = 1/2$ and predicts equal fractions of each N_2O_5 isotope. The highest observed $Cl^{15}NO_2$ fraction is 0.8 for 3.7 M NO_3^- in Figure 4. This isotope fraction yields $y = 1/8$ and predicts that $^{14,14}N_2O_5$ = 11%, $^{14,15}N_2O_5$ = 18%, and $^{15,15}N_2O_5$ = 71% mole percent. These isotopic N_2O_5 fractions were confirmed by direct Kinetic Multi-Layer calculations. In general, the equations above show that high $Cl^{15}NO_2$ isotope fractions imply remarkably extensive NO_3^- exchange prior to irreversible hydrolysis or chlorination, making $^{15,15}N_2O_5$ the majority species for $Cl^{15}NO_2$ isotope fractions greater than 0.64 ($y > 0.28$).

VI. Different Activation and Deactivation Steps in N_2O_5 Chlorination and Nitration

We ask what happens when the activation and deactivation steps are different for different reactions, this time for the simpler case of the relative rates of chlorination and nitration. We consider a restricted reaction set in which only $^{14,14}N_2O_5$* is involved and hydrolysis is excluded. We start with $^{14,14}N_2O_5$ that is activated to two different states, a and b, which represent unique N_2O_5 excitation for chlorination (a) and nitration (b). In this case, the four pertinent reactions are:

$^{14,14}N_2O_5 \rightarrow {}^{14,14}N_2O_5^a$	k_a (forward) and k'_a (backward)	^{14,14}N activation for chlorination
$^{14,14}N_2O_5 \rightarrow {}^{14,14}N_2O_5^b$	k_b (forward) and k'_b (backward)	^{14,14}N activation for nitration
$^{14,14}N_2O_5^a + Cl^- \rightarrow Cl^{14}NO_2 + {}^{14}NO_3^-$	k_{Cl^-}	^{14}N chlorination from state a
$^{14,14}N_2O_5^b + {}^{15}NO_3^- \rightarrow {}^{14,15}N_2O_5 + {}^{14}NO_3^-$	$k_{NO_3^-}$ (or k_{exd})	^{14}N nitration from state b

Upon applying the steady-state approximation to $[^{14,14}N_2O_5^a]$ and $[^{14,14}N_2O_5^b]$, we obtain the ratio of $Cl^{14}NO_2$ production rate/$^{14,15}N_2O_5$ production rates to be

$$d[Cl^{14}NO_2]/dt/d[^{14,15}N_2O_5]/dt = (k_{Cl^-}/k_{NO_3^-})[Cl^-]/[^{15}NO_3^-]$$

$$\times (k_a/k_b) \times (k'_b + k_{NO_3^-}[^{15}NO_3^-]) / (k'_a + k_{Cl^-}[Cl^-])$$

The production ratio now depends on the individual activation and deactivation rate constants. These constants cannot unfortunately be determined from our data, and we do not pursue this analysis further. When state a = state b, there is a single activated state $N_2O_5^*$, $k_a = k_b$ and $k'_a = k'_b$, the sums in the numerator and denominator become a common term $k' + k_{Cl^-}[Cl^-] + k_{NO_3^-}[^{15}NO_3^-]$, and the expression reduces to just $(k_{Cl^-}/k_{NO_3^-})[Cl^-]/[^{15}NO_3^-]$. In this case, Cl^- and NO_3^- react with the same N_2O_5* species, and the way in which N_2O_5* is produced or deactivated does not affect the ratio of $Cl^{14}NO_2/^{14,15}N_2O_5$ production rates or the ratio $k_{Cl^-}/k_{NO_3^-}$.

VII. Equivalence of N_2O_5* and NO_2^+ When NO_3^- Exchange Is the Only Deactivation Step

In Table S1, reactions 4-7 and 16-19 are NO_3^- exchange reactions with N_2O_5*, all assigned the same rate constant. This table follows the S_N2 mechanism. The labeled N_2O_5* may be viewed equivalently as an S_N1 NO_2^+ species or as an S_N2 molecular species when deactivation occurs solely by chemical exchange with NO_3^-. Reactions 1-3, for example, become ${}^{x,y}N_2O_5 \rightarrow {}^{x}NO_2^+ + {}^{y}NO_3^-$ in the S_N1 NO_2^+ mechanism and reactions 4-7 and 16-19 become the deactivation reaction ${}^{x}NO_2^+ + {}^{y}NO_3^- \rightarrow {}^{x,y}N_2O_5$. This equivalence can be seen by starting with reaction 4:

$${}^{14,14}N_2O_5^* + {}^{15}NO_3^- \rightarrow {}^{14,15}N_2O_5 + {}^{14}NO_3^- \qquad \text{(reaction 4)}$$

We first view ${}^{14,14}N_2O_5$* as the reactive species composed of ${}^{14}NO_2^+$ and charged-paired ${}^{14}NO_3^-$ ions that are written as separate species. This connection bridges the two mechanisms:

$${}^{14}NO_2^+ + {}^{14}NO_3^- + {}^{15}NO_3^- \rightarrow {}^{14,15}N_2O_5 + {}^{14}NO_3^- \qquad \text{(rewritten reaction 4)}$$

After exchange, the product N_2O_5 is unactivated and remains molecular. We next cancel the spectator ${}^{15}NO_3^-$ ions to obtain the S_N1 NO_2^+ recombination-deactivation step:

$${}^{14}NO_2^+ + {}^{15}NO_3^- \rightarrow {}^{14,15}N_2O_5$$

In both S_N1 and S_N2 mechanisms, ${}^{15}NO_3^-$ must diffuse to the activated species, N_2O_5* or NO_2^+, in order to deactivate these species. Thus, the two species can be used interchangeably when NO_3^- is the only species that can deactivate N_2O_5* or NO_2^+. A key distinction between these two species is that N_2O_5* can spontaneously deactivate, while NO_2^+ must recombine with NO_3^- to deactivate. As shown throughout this SI, however, the final kinetic expressions for the $ClNO_2$ isotope fraction and the $k_{Cl^-}/k_{NO_3^-}$ rate constant ratio do not depend on the activation or deactivation steps.

VIII. Resistor Models for N_2O_5 Reactive Uptake in Relation to Figures 8 and S6

To predict reactive uptake in Figure 8 of the main text, we use a resistor model that accounts for the finite size of the measured aerosol particles. This model bridges diffusion and reaction of a gas molecule into a region that is small with respect to particle radius and uniform filling of the particle by the gas. It is given by Hanson et al.[18]

$$\frac{1}{\gamma} = \frac{1}{\alpha} + \frac{<v>}{4HRT\sqrt{k_{\mathrm{h}}D}} * \frac{1}{\left(\coth(q) - \frac{1}{q}\right)} \qquad \mathrm{S-5}$$

where γ is the reactive uptake coefficient (0 to 1), α is the probability (0 to 1) that an impinging N_2O_5 molecule enters the liquid, $<v> = (8RT/\pi m)^{1/2}$ is its average speed, H is the Henry's Law constant of N_2O_5 in M/atm, D is its diffusion coefficient in solution, and k_h is the first-order rate constant for the irreversible hydrolysis of N_2O_5 in the presence of NO_3^-. The coth term depends on $q = r/L$, where L is the reacto-diffusive length, $(D/k_h)^{1/2}$, and r is the particle radius. This term accounts for the finite size of the particle, but there is no interfacial component in this model.

Equation S-5 spans two limits for $q \ll 1$ and $q \gg 1$. When the droplet radius is much larger than the reaction depth and reaction occurs near the surface, $q \gg 1$, the term $\coth(q) - 1/q$ approaches 1, and eq S-5 reduces to near-surface diffusion-limited uptake:

$$\frac{1}{\gamma} = \frac{1}{\alpha} + \frac{<v>}{4HRT\sqrt{k_{\mathrm{h}}D}} \qquad \text{near}-\text{surface diffusion limit} \qquad \mathrm{S-6}$$

For reference, $\coth(q) - 1/q$ approaches 0.85 as q approaches 7. In the opposite case in which $r \ll L$ and q approaches 0, reaction occurs throughout the volume V of a spherical droplet by gas transport across surface area S, and eq S-5 becomes:

$$\frac{1}{\gamma} = \frac{1}{\alpha} + \frac{<v>}{4HRT\sqrt{k_{\mathrm{h}}D}}\frac{S}{V} \qquad \text{volume filling} \qquad \mathrm{S-7}$$

where $S/V = 3/r$ and $\coth(q) - 1/q = q/3$. In practical terms, $\coth(q) - 1/q$ is 15% below q/3 for q =

1.7. The intermediate regime between q = 1.7 and 7 is best described by eq S-5.

In the presence of NO_3^-, k_h depends on the nitrate concentration (nitrate effect):[3,8,9,15]

$$k_h = k_{pure}\left(1 - \frac{k_{NO_3^-}[NO_3^-]}{k_{NO_3^-}[NO_3^-] + k_w[H_2O]}\right)$$

or

$$k_h = \frac{k_{pure}}{1 + \frac{k_{NO_3^-}[NO_3^-]}{k_w[H_2O]}} \qquad S-8$$

where k_{pure} is the pure water hydrolysis rate constant in the absence of NO_3^-, equal to the activation rate constant to create N_2O_5* or NO_2^+. k_h and therefore γ decrease with increasing $[NO_3^-]$. This expression is obtained by applying the steady-state approximation to N_2O_5* or NO_2^+ when deactivation solely occurs by nitrate exchange (in order to use the same expression for the N_2O_5* or NO_2^+ intermediate, as stated in section V).

We used eqs S-7 and S-8 to create Figure 8A in the volume-filling limit using the parameters and analysis in Bertram and Thornton[3] (where the $1/\alpha$ is left out to reduce the parameter set even further because the reaction probability is so much smaller than the entry probability). This data set is H = 2.1 M/atm, V/S = 3.75×10^{-6} cm, and k_{pure} = 1.15×10^{6}/s. These values generate uptake γ(0 M $NaNO_3$) = 0.036. As seen in Figure 8, the key parameter that controls the shape of the curve for a fixed particle radius is the rate constant ratio $k_{NO_3^-}/k_w$, which is 17 for Bertram and Thornton[3] and 80-230 from our studies. Our higher rate constant ratios generate a poor fit to the data.

Incorporating Spontaneous N_2O_5* Deactivation and Fitting Figure 8A

Equation 8 equation can be modified to include **s**pontaneous (water-driven) **d**eactivation of N_2O_5* with rate constant k_{sd}, reaction R-8 of the main text, using the S_N2 deactivation model described above. The explicit mechanism for unlabeled N_2O_5 (for which there is no role for nitrate exchange) is:

$N_2O_5 \rightarrow N_2O_5^*$	spontaneous (water-induced) activation k_{act} (R8)
$N_2O_5^* \rightarrow N_2O_5$	spontaneous (water-induced) deactivation k_{sd} (R-8)
$N_2O_5^* + NO_3^- \rightarrow N_2O_5 + NO_3^-$	nitrate-induced deactivation k_{nd} (R11)
$N_2O_5^* + H_2O \rightarrow 2\ H^+ + 2\ NO_3^-$	hydrolysis k_w (R9)
$N_2O_5^* + Cl^- \rightarrow ClNO_2 + NO_3^-$	chlorination k_{Cl^-} (R10)

In the S_N1 NO_2^+ mechanism, nitrate-induced deactivation k_{nd} becomes NO_2^+ + NO_3- recombination k_{rec}. This is sometimes labeled as $k_{NO_3^-}$. The spontaneous deactivation step R-8 does not exist in the S_N1 mechanism, but it must be present in the S_N2 mechanism as the reverse of the activation step R8. We do not include activation by ions or deactivation by ions other than NO_3^- in this mechanism (pages S13 and S14) in order to limit the number of new parameters to just k_{sd}. Lastly, we note that, in an experiment that does not resolve isotopes, k_{nd} is the sum of nitrate-induced reactive and not reactive deactivation, k_{nrd} and k_{exd} (on pages S13 and S14).

The steady-state solution for k_h with spontaneous deactivation is:

$$\mathrm{k_h(spon\ deac)} = \frac{k_{\mathrm{pure}}}{1 + \frac{k_{\mathrm{NO_3^-}}[\mathrm{NO_3^-}]}{k_{\mathrm{w}}[\mathrm{H_2O}]} + \frac{k_{\mathrm{sd}}}{k_{\mathrm{w}}[\mathrm{H_2O}]}} \qquad \mathrm{S-9}$$

This equation contains the additional fitting parameter, k_{sd}/k_w. It is now possible to fit the data more closely in Figure 8A, as shown in Figure S6 below. We use our middle value of $k_{NO_3^-}/k_w$ =

150 and constrain the 0 M $NaNO_3$ uptake value to be 0.036, as above (requiring k_{pure} to be 7.35×10^6). We find that k_{sd}/k_w = 300 M leads to a fit that closely matches the data. Thus, the ratio ($k_{sd}/(k_w[H_2O])$) is roughly 5-6. For 1 M NO_3^- and Cl^- and 54 M H_2O, $k_{sd}/k_w[H_2O]$ is 5.5 compared to 2.7 for $(k_{NO_3^-}/k_w)[NO_3^-]/[H_2O]$ and 11 for $(k_{Cl^-}/k_w)[Cl^-]/[H_2O]$. Thus, nitration is half as fast as water-driven deactivation and chlorination is twice as fast for 1 M salt solutions. We do not have sufficient confidence to judge whether this interpretation is meaningful and provides support for the S_N2 model with spontaneous N_2O_5* deactivation, or if inclusion of the k_{sd} parameter leads to a better fit just because it is an additional fitting parameter. We do hope, however, that this analysis helps to constrain further theory efforts of N_2O_5 reactivity in concentrated salty water.

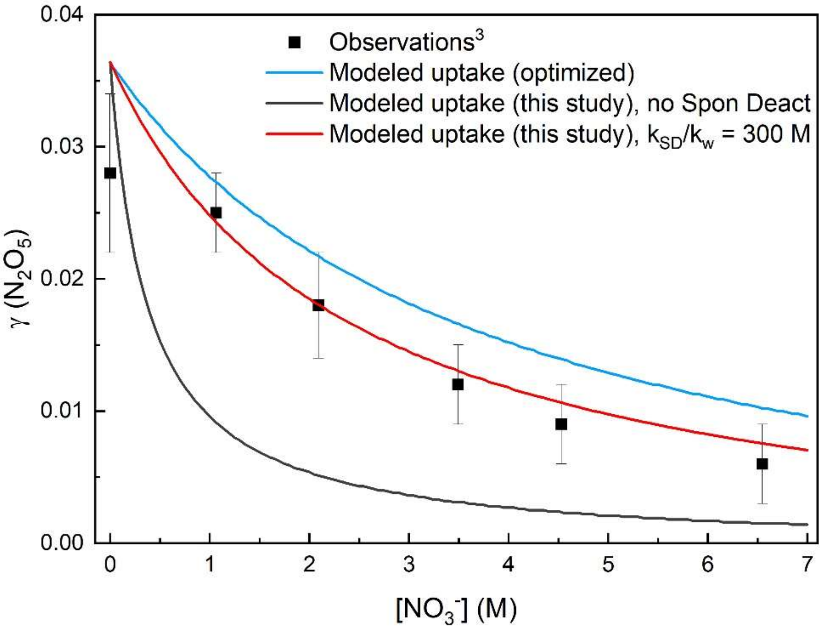

Figure S6. N_2O_5 reactive uptake versus added nitrate. The data and blue and red curves are reproduced from Figure 8A of the main text. The data is from Bertram and Thornton,[3] as is the S_N1 NO_2^+ fit in blue using $k_{NO_3^-}/k_w$ = 17. The gray fit is our S_N2 model with $k_{NO_3^-}/k_w$ = 150 without spontaneous deactivation. The red fit is with spontaneous deactivation with rate constant ratio k_{sd}/k_w = 300 M (where the dimensional ratio is $k_{sd}/k_w[H_2O]$) .

Mixed Deactivation/Activation Model

We can alternatively fit the data in Figure S6 by hypothesizing that nitrate exchange is accompanied by deactivation of N_2O_5* in only a fraction of the exchange events, while it maintains N_2O_5* otherwise. This fractional deactivation may be incorporated into k_h by multiplying $k_{NO_3^-}/k_w$ by a probability f_{deac}, which spans 0 to 1. One possible scenario may invoke an interfacially located N_2O_5 that is activated by its limited solvation, as postulated by Jin and Limmer.[13] In this picture, f_{deac} reflects the time scales for nitrate exchange versus transport from the interfacial region into the bulk region, where N_2O_5 reactivity is reduced. We incorporate this reduction by modifying eq S-8 as:

$$\mathrm{k_h(partial\ deactivation)} = \frac{k_{\mathrm{pure}}}{1 + \frac{f_{\mathrm{deac}} k_{\mathrm{NO_3^-}} [\mathrm{NO_3^-}]}{k_{\mathrm{w}} [\mathrm{H_2O}]}} \qquad \mathrm{S-8'}$$

Our value of $k_{NO_3^-}/k_w$ = 150 must be reduced to $k_{NO_3^-}/k_w$ = 17 in order to transform the gray curve in Figure S6 into the blue curve. This reduction is achieved by setting f_{deac} to 17/150 = 0.11. A slightly better fit (not shown) is obtained when f_{deac} = 0.20, which corresponds to $k_{NO_3^-}/k_w$ = 30 in ref 15. These f_{deac} values imply that nitrate exchange occurs more often than N_2O_5 deactivation, perhaps because nitrate exchange in most events is not accompanied by N_2O_5 transport from the interfacial to the bulk region. We note that k_{sd} introduced in eq S-9 is set to zero in this model. This assumption may be unphysical in that spontaneous transport of N_2O_5 across the interface should occur as well. We include this alternative model for completeness and hope that a full theoretical analysis will reveal its relevance.

IX. Parameterizing NO_3^-, Cl^-, and H_2O Concentrations from the Aerosol Inorganics Model (E-AIM)

The different analyses of solution concentrations in our studies and those of Bertram and

Thornton[3] require tabulations of [NO_3^-], [Cl^-], and [H_2O] from 0 to 7 M $NaNO_3$ and 0 to 3 M NaCl. Our solutions are prepared volumetrically and so the NO_3^- and Cl^- concentrations are known. The H_2O concentration is determined by using the E-AIM program/Aqueous Solutions to compute solution volumes.[19-21] In the case of $NaNO_3$ dissolved in H_2O without NaCl, Figure S7 shows a nearly straight line fit to the E-AIM/Aqueous solution output at 298.15 K yields [H_2O] = 55.669 – 1.933[NO_3^-]. The error in the y-intercept is 0.32 M.

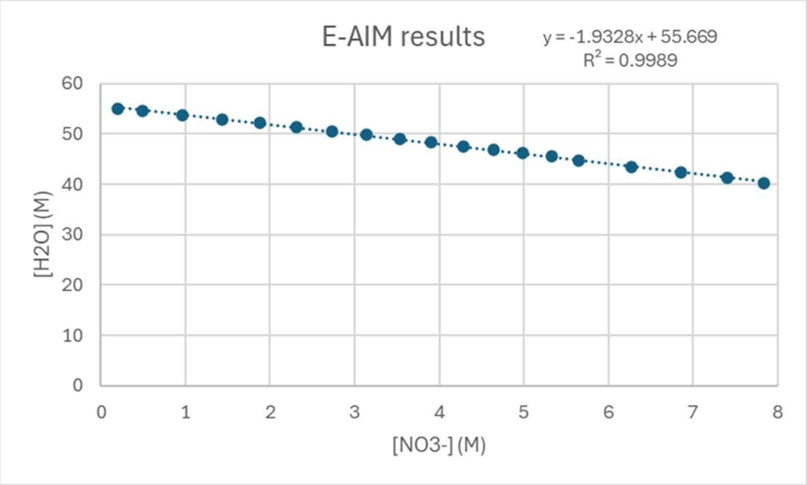

Figure S7. Empirical relation between [H_2O] and [NO_3^-] using the E-AIM program[19-21] with Model 3 and Aqueous Solution mode. Blue dots are explicit results from E-AIM using a range of inputted $NaNO_3$ values (0.2 to 10.81 molal). The dashed blue line is the linear regression, which yields the equation [H_2O] = 55.669 – 1.933[NO_3^-].

Chloride and nitrate ion concentrations in aerosol droplets are also needed to analyze data from Bertram and Thornton[3] and to construct Figure 8B of the main text. The data are reported at specified wt% solutes in the prepared solutions that are then equilibrated as droplets at a specified relative humidity. To correlate [Cl^-], [NO_3^-], and [H_2O], we convert these initial NaCl and $NaNO_3$ wt% values into moles and then use E-AIM/Simple Calculations to compute the volumes, H_2O moles, and ion and H_2O molarities.[19-21]

X. Recasting Rate Constants in Terms of Activities Instead of Molarities

Reaction rates involving ions are often usefully expressed in terms of activities instead of molarities.[22] Rate constants k multiplying molarities [X] yield a rate k[X] that may be recast using activities $k'a_X = k'\, y^{\pm}[X]$, where $y^{\pm}$ is the mean molarity-scale ionic activity coefficient. Thus, k' equals $k/y^{\pm}$ to obtain the same rate at the same molarity. The activity coefficients $y_{\pm}$ for NaCl and $NaNO_3$ may be obtained using eqs 2.12 and 2.23 in ref 23 and the individual ion mole-faction activity coefficients $f_{\pm}$ generated by E-AIM,[19-21] where ion concentrations are entered as molalities. Interconversions between molality and molarity are given by eq 2.20 in ref 23. The mass density is obtained from the E-AIM output. All activity coefficients were determined in H_2O, although the experiments were performed in D_2O.

The $Cl^{15}NO_2$ isotope fractions only depend on ratios of the NaCl and $Na^{15}NO_3$ rate constants (Table 1 of the main text). We therefore compare $(k_{\text{NaCl}}/k_{\text{NaNO3}})$ and $(k'_{\text{NaCl}}/k'_{\text{NaNO3}}) = (k_{\text{NaCl}}/k_{\text{NaNO3}})/(y^{\pm}_{\text{NaCl}}/y^{\pm}_{\text{NaNO3}})$. The usefulness of the activity scale may be gauged by comparing these ratios at low and high salt concentrations, chosen for testing to be 0.1 M and 3.26 M each:

1) 0.1 M NaCl + 0.1 M $NaNO_3$ (0.101 molal)

E-AIM $y^{\pm}$ values are 0.728 (NaCl) and 0.714 ($NaNO_3$) and $y^{\pm}_{\text{NaCl}}/y^{\pm}_{\text{NaNO3}}$ = 1.02

molarity scale $(k_{\text{NaCl}}/k_{\text{NaNO3}})$ = 4.9 (average value of 3.6-6.2 from Table 1 of main text)

activity scale $(k'_{\text{NaCl}}/k'_{\text{NaNO3}})$ = 4.9/1.02 = 4.8.

This 2% change is well within the uncertainty of our rate constant ratio measurements.

3) 3.26 M NaCl + 3.26 M $NaNO_3$ (each 4.0 molal)

E-AIM $y^{\pm}$ values are 1.063 (NaCl) and 0.546 ($NaNO_3$) and $y^{\pm}_{\text{NaCl}}/y^{\pm}_{\text{NaNO3}}$ = 1.95

molarity scale $(k_{\text{NaCl}}/k_{\text{NaNO3}})$ = 3.6 (average value of 2.2-5.0 from Table 1 of main text)

activity scale $(k'_{\text{NaCl}}/k'_{\text{NaNO3}})$ = 3.6/1.95 = 1.8.

The rate constant ratio is halved on the activity scale in comparison to the molarity scale due to the smaller activity coefficient of $NaNO_3$ then of NaCl. This comparison leads to the intriguing implication that chloride and nitrate attack have more similar rate constants at higher concentrations, as part of the difference is now incorporated into the activity coefficient ratio. The distinct change from the 0.1 M solution can be attributed to stronger interactions among the ions and a greater number of shared water molecules in the ion hydration shells in the more concentrated solutions.[22, 23] We note that the change in NaCl/$NaNO_3$ rate constant ratio in Table 1 is smaller for the molarity-based values (4.9 to 3.6) than for activity-based value (4.8 to 1.8). This difference suggests that, if the two rate constant ratios are to apply over a wide concentration range, it seems more accurate to use molarities than activities. It would be fruitful to compare activity coefficient ratios over a wide range of concentrations and ions, as the changes in activity coefficients are not monotonic and can vary greatly for different salts and acids (see Figure 9.3 of ref 23).

We may also ask if solvent water activities are more appropriate than water molarities for our experiments. Solvent activities are commonly expressed on the mole fraction scale, for which $a_w = P_w/P^o_w$ = relative humidity, where P_w is the water vapor pressure. For 0.1 M NaCl + 0.1 M $NaNO_3$, a_w = 0.993, and for 3.26 NaCl + 3.26 M $NaNO_3$, a_w = 0.739. These water activities are close to 1 and very different from the 40 to 55 M H_2O molarities shown in Figure S7. The use of water activities transforms a second-order rate expression for hydrolysis of N_2O_5 of $k[H_2O][N_2O_5]$ into an essentially first-order expression $k''a_{N2O5}a_w$, and gives a very different numerical value for k''. To maintain the second-order nature of the rate constant, we judge it to be more appropriate to use water molarities instead of activities. The molarity-based activity coefficients for water do not affect the molarity scale much, as these activity coefficients each close to one: 1.008 and 0.957, respectively, for the 0.1/3.26 M NaCl + 0.1/3.26 M $NaNO_3$ solutions.

References

(1) Morrison, G.; Lakey, P. S. J.; Abbatt, J.; Shiraiwa, M. Indoor Boundary Layer Chemistry Modeling. *Indoor Air* **2019**, *29*, 956–967. https://doi.org/10.1111/ina.12601.

(2) Shiraiwa, M.; Pfrang, C.; Poschl, U. Kinetic Multi-Layer Model of Aerosol Surface and Bulk Chemistry (KM-SUB): The Influence of Interfacial Transport and Bulk Diffusion on the Oxidation of Oleic Acid by Ozone. *Atmos Chem Phys* **2010**, *10*, 3673-3691, https://doi.org/10.5194/acp-10-3673-2010.

(3) Bertram, T. H.; Thornton, J. A. Toward a General Parameterization of N_2O_5 Reactivity on Aqueous Particles: The Competing Effects of Particle Liquid Water, Nitrate and Chloride. *Atmos Chem Phys* **2009**, *9*, 8351-8363, https://doi.org/10.5194/acp-9-8351-2009.

(4) Kregel, S. J.; Derrah, T. F.; Moon, S.; Limmer, D. T.; Nathanson, G. M.; Bertram, T. H. Weak Temperature Dependence of the Relative Rates of Chlorination and Hydrolysis of N_2O_5 in NaCl–Water Solutions. *J. Phys. Chem. A* **2023**, *127*, 1675–1685. https://doi.org/10.1021/acs.jpca.2c06543.

(5) Behnke, W.; George, C.; Scheer, V.; Zetzsch, C. Production and Decay of $ClNO_2$ from the Reaction of Gaseous N_2O_5 with NaCl Solution: Bulk and Aerosol Experiments. *J. Geophys. Res. Atmospheres* **1997**, *102* (D3), 3795–3804. https://doi.org/10.1029/96JD03057.

(6) Cruzeiro, V. W. D.; Galib, M.; Limmer, D. T.; Götz, A. W. Uptake of N_2O_5 by Aqueous Aerosol Unveiled Using Chemically Accurate Many-Body Potentials. *Nat. Commun.* **2022**, *13*, 1266. https://doi.org/10.1038/s41467-022-28697-8.

(7) Tang, M. J.; Cox, R. A.; Kalberer, M. Compilation and Evaluation of Gas Phase Diffusion Coefficients of Reactive Trace Gases in the Atmosphere: Volume 1. Inorganic Compounds. *Atmos Chem Phys* **2014**, *14*, 9233–9247.

(8) Mentel, T. F.; Sohn, M.; Wahner, A. Nitrate Effect in the Heterogeneous Hydrolysis of

Dinitrogen Pentoxide on Aqueous Aerosols, **1999**, *1*, 5451-5457, https://doi.org/10.1039/A905338G.

(9) Gržinić, G.; Bartels-Rausch, T.; Türler, A.; Ammann, M. Efficient Bulk Mass Accommodation and Dissociation of N_2O_5 in Neutral Aqueous Aerosol. *Atmospheric Chem. Phys.* **2017**, *17* (10), 6493–6502. https://doi.org/10.5194/acp-17-6493-2017.

(10) Hirshberg, B.; Rossich Molina, E.; Götz, A. W.; Hammerich, A. D.; Nathanson, G. M.; Bertram, T. H.; Johnson, M. A.; Gerber, R. B. N_2O_5 at Water Surfaces: Binding Forces, Charge Separation, Energy Accommodation and Atmospheric Implications. *Phys. Chem. Chem. Phys.* **2018**, *20* (26), 17961–17976. https://doi.org/10.1039/C8CP03022G.

(11) Karimova, N. V.; Chen, J.; Gord, J. R.; Staudt, S.; Bertram, T. H.; Nathanson, G. M.; Gerber, R. B. S_N2 Reactions of N_2O_5 with Ions in Water: Microscopic Mechanisms, Intermediates, and Products. *J. Phys. Chem. A* **2020**, *124* (4), 711–720. https://doi.org/10.1021/acs.jpca.9b09095.

(12) Galib, M.; Limmer, D. T. Reactive Uptake of N_2O_5 by Atmospheric Aerosol Is Dominated by Interfacial Processes. *Science* **2021**, *371* (6532), 921–925. https://doi.org/10.1126/science.abd7716.

(13) Moon, S.; Limmer, D. T. Enhanced $ClNO_2$ Formation at the Interface of Sea-Salt Aerosol. *J. Phys. Chem. Lett.* **2024**, *15* (37), 9466–9473. https://doi.org/10.1021/acs.jpclett.4c02289.

(14) Gaston, C. J.; Thornton, J. A. Reacto-Diffusive Length of N_2O_5 in Aqueous Sulfate- and Chloride-Containing Aerosol Particles. *J. Phys. Chem. A* **2016**, *120* (7), 1039–1045. https://doi.org/10.1021/acs.jpca.5b11914.

(15) Griffiths, P. T.; Badger, C. L.; Cox, R. A.; Folkers, M.; Henk, H. H.; Mentel, T. F. Reactive Uptake of N_2 O_5 by Aerosols Containing Dicarboxylic Acids. Effect of Particle Phase,

Composition, and Nitrate Content. *J. Phys. Chem. A* **2009**, *113* (17), 5082–5090. https://doi.org/10.1021/jp8096814.

(16) Wahner, A.; Mentel, T. F.; Sohn, M.; Stier, J. Heterogeneous Reaction of N_2O_5 on Sodium Nitrate Aerosol. *J. Geophys. Res. Atmospheres* **1998**, *103* (D23), 31103–31112. https://doi.org/10.1029/1998JD100022.

(17) Gaston, C. J.; Thornton, J. A. Reacto-Diffusive Length of N_2O_5 in Aqueous Sulfate- and Chloride-Containing Aerosol Particles. *J Phys Chem A* **2016**, *120*, 1039–1045. https://doi.org/10.1021/acs.jpca.5b11914.

(18) Hanson, D. R.; Ravishankara, A. R.; Solomon, S. Heterogeneous Reactions in Sulfuric Acid Aerosols: A Framework for Model Calculations. *J. Geophys. Res. Atmospheres* **1994**, *99* (D2), 3615–3629, https://doi.org/10.1029/93JD02932.

(19) Clegg, S. L.; Brimblecombe, P.; Wexler, A. S. A Theromdynamic Model of the System H^+ - NH_4^+ - SO_4^{2-} - NO_3^- - Cl^- - H_2O at 298.15 K. *J Phys Chem A* **1998**, *102*, 2155–2171.

(20) Wexler, A. S.; Clegg, S. L. Atmospheric aerosol models for systems including the ions H^+, NH_4^+, Na^+, SO_4^{2-}, NO_3^-, Cl^-, Br^- and H_2O. *J. Geophys. Res.* **2002**, *107*, art. no. 4207.

(21) E-AIM website: https://www.aim.env.uea.ac.uk/aim/aim.php.

(22) Laidler, K. J *Chemical Kinetics*, 3rd ed., **1987,** Pearson, Chapter 6.

(23) Robinson, R. A.; Stokes, R. H. Electrolyte Solutions, 2nd ed., **1959**, Chapter 2.
